# Phenolics from Medicinal and Aromatic Plants: Characterisation and Potential as Biostimulants and Bioprotectants

**DOI:** 10.3390/molecules26216343

**Published:** 2021-10-20

**Authors:** Musa Kisiriko, Maria Anastasiadi, Leon Alexander Terry, Abdelaziz Yasri, Michael Henry Beale, Jane Louise Ward

**Affiliations:** 1 Plant Science Laboratory, Cranfield University, Cranfield MK43 0AL, UK; musa.Kisiriko@cranfield.ac.uk (M.K.); m.anastasiadi@cranfield.ac.uk (M.A.); l.a.terry@cranfield.ac.uk (L.A.T.); 2 AgroBioSciences Research Division, Mohammed VI Polytechnic University, Lot 660, Moulay Rachid, Ben Guerir 43150, Morocco; aziz.yasri@um6p.ma; 3 Rothamsted Research, West Common, Harpenden AL5 2JQ, UK; mike.beale@rothamsted.ac.uk

**Keywords:** biostimulant, bioprotectant, phenolic, medicinal, aromatic

## Abstract

Biostimulants and bioprotectants are derived from natural sources and can enhance crop growth and protect crops from pests and pathogens, respectively. They have attracted much attention in the past few decades and contribute to a more sustainable and eco-friendly agricultural system. Despite not having been explored extensively, plant extracts and their component secondary metabolites, including phenolic compounds have been shown to have biostimulant effects on plants, including enhancement of growth attributes and yield, as well as bioprotectant effects, including antimicrobial, insecticidal, herbicidal and nematicidal effects. Medicinal and aromatic plants are widely distributed all over the world and are abundant sources of phenolic compounds. This paper reviews the characterisation of phenolic compounds and extracts from medicinal and aromatic plants, including a brief overview of their extraction, phytochemical screening and methods of analysis. The second part of the review highlights the potential for use of phenolic compounds and extracts as biostimulants and bioprotectants in agriculture as well as some of the challenges related to their use.

## 1. Introduction

The increasing world population necessitates the agricultural sector to develop sustainablesystems that can produce enough food for the growing numbers of people. This entails both producing more food and protecting what is currently being produced. To date, efforts to boost food production have led to heavy reliance on mineral fertilisers and synthetic chemicals for improving yields and protecting crops from pathogens and pests to boost food production and reduce crop loss [1]. However, there is increasing advocacy for the reduction of use of synthetic fertilisers and pesticides to achieve a more sustainable and ecofriendly agricultural ecosystem. In the past few decades, biostimulants and bioprotectants have gained appreciable recognition as both are derived from natural sources and have proven to be a possible solution [2,3].

Biostimulants are substances derived from natural sources that improve plant growth and yield by activating physiological and metabolic processes to improve nutrient uptake and to relieve plants of effects resulting from abiotic stresses [3,4]. Unlike fertilisers, biostimulants do not directly provide plants with nutrients. They instead enhance the uptake of nutrients, hence reducing the need for the application of fertilisers [5]. The primary sources of biostimulants include microorganisms (*viz.* bacteria, yeasts and fungi), algae (mainly seaweed), protein hydrolysates and amino acids, chitin and chitosan derivatives, humic substances, waste and compost extracts, and extracts from higher plant parts (seeds, leaves and roots) [2]. Seaweed extracts, especially those from the brown algae *Ascophyllum nodosum*, are commercially available and have achieved increasing and extensive use in agriculture due to their significant biostimulant effects on plant nutrient uptake, stress tolerance and produce quality [6]. Bioprotectants are substances derived from natural sources that have the potential to prevent crops from being attacked by pests and pathogens. Several plant-derived extracts have been shown to effectively manage pests and pathogens during growth and after harvesting crops through toxic and inhibitive mechanisms [7,8].

The biostimulant and bioprotectant effects have largely been ascribed to the secondary metabolites contained in plant extracts. Unlike the primary metabolites, secondary metabolites are not involved in intrinsic life functions like growth and development. They are important for the adaptation of plants to their environments, for instance, defence and protection under stress conditions, and their distribution varies among plant species. There are various classifications of secondary metabolites but generally, the main groups are alkaloids, terpenes and terpenoids and phenolic compounds [9,10,11]

Phenolic compounds (used interchangeably with ‘phenolics’ henceforth) are arguably the most abundant secondary metabolites in plants and are often found in the cell walls and vacuoles of epidermal and subepidermal cells [12]. These phenolics can be constitutive or induced in plants. Constitutive phenolics are pre-formed in plants, usually being synthesized during normal growth and development, whereas induced phenolics are synthesized in response to biotic and abiotic stresses. Induced phenolics can also be constitutive, but their synthesis and accumulation are increased by exposure of the plant to biotic and abiotic stresses. Examples of induced phenolic compounds are the phytoalexins, e.g., resveratrol, that are induced in plants in response to pathogen attack to play an antimicrobial role [12,13]. The roles of endogenous phenolic compounds in plants are very diverse. They are antioxidants that scavenge reactive oxygen species; they are accumulated in epidermal cells to act as screens against damaging UV radiation; they defend plants against herbivores, pathogens and weeds; they enhance the mobilization and uptake of nutrients; they are implicated in seed germination and dormancy; phenolics especially anthocyanins act as pigments that attract pollinators and seed dispersal agents, *et cetera* [10,11,12,13,14]. A recent review by Sharma et al. (2019) elaborated the response and role of endogenous phenolics in the tolerance of plants towards abiotic stress factors like heavy metals, drought, salinity, UV light and heat among others [14]. Lattanzio et al. (2006) comprehensively reviewed the roles of endogenous phenolics in the resistance mechanisms of plants towards fungal pathogens and insect pests [12]. Mandal et al. (2010) highlighted the roles of phenolic acids as signaling molecules in plant–microbe symbioses [15].

Medicinal and aromatic plants are some of the most prominent sources of phenolic compounds, and their phytochemical constituents often have therapeutic value or can be precursors for the development of pharmaceuticals. They have been used worldwide since prehistoric times for treating several ailments and still find increasing use today in herbal medicine formulations as well as alternative and complementary medicine. Indeed, medicinal plants are still widely used in ayurvedic and Chinese traditional medicines [10]. However, despite this wide usage, there is a lack of clinical evidence for the pharmacological effects attributed to many of these medicinal plants. Aromatic plants are common culinary additives that are often used to flavour and season food. They are also used in perfumes and cosmetics because they are odorous and many of them are also potent medicinal sources. Therefore, the term medicinal plant is also commonly found in literature referring to aromatic plants. In fact, many plants are jointly referred to as “medicinal and aromatic plants”. The therapeutic properties of medicinal and aromatic plants are related to the fact that most of their component compounds, including phenolics are largely antioxidants that can protect the body against oxidative stress and prevent the incidence of many diseases, including cancers, inflammation and cardiovascular diseases [16,17]. Throughout the course of this review, we shall cover medicinal and aromatic plants that have been used in various characterisation and biostimulant or bioprotectant assay studies. Some of these will be represented in the body of the text and majority will be summarised in various tables in the paper.

Exogenously applied extracts, and phenolic compounds derived from medicinal and aromatic plants have been assessed for their biostimulant and bioprotectant properties in plants. They have shown biostimulant activities, such as enhancing seed germination, rooting, shooting and fruiting, as well as bioprotectant activities, including antimicrobial, insecticidal, nematicidal and herbicidal activities [18,19,20,21,22]. Extraction of plant material with polar solvents such as water, methanol or hydro-alcoholic solvents results in extracts that largely contain phenolic compounds [23]. Although it is argued that the bioactivity of these extracts is due to synergistic interactions between the components, it has also been shown that a large proportion of the effects can be attributed to the phenolic compounds [20,22]. Biostimulant and bioprotectant activities have also been shown in individual isolated phenolic compounds and this reinforces the fact that they can be bioactive on their own or work in synergy with other compounds.

Therefore, the goal of this paper is to review the characterisation of phenolic compounds and plant extracts from medicinal and aromatic plants and showcase the potential for their use as biostimulants and bioprotectants in agriculture when applied exogenously. As well as individual compounds, plant extracts containing mixtures of components are equally important. This is especially the case for extracts made with polar solvents (e.g., water) where phenolic compounds are often abundant. The study of extracts, especially those made with water, is particularly important because this is the most economically feasible way that farmers mainly in poor communities would use them. Most plants can readily be found growing on their own and can easily be obtained, crushed, mixed with water and applied to crops. Since most phenolic compounds naturally occur in medicinal and aromatic plants, this review will also include studies that used commercial phenolic compounds that were not necessarily obtained from medicinal and aromatic plants. The review shall also highlight some of the gaps and challenges related to the application of these phenolic compounds and plant extracts, including issues related to efficacy and scaling.

## 2. Phenolic Composition of Medicinal and Aromatic Plants

**Figure 1:**
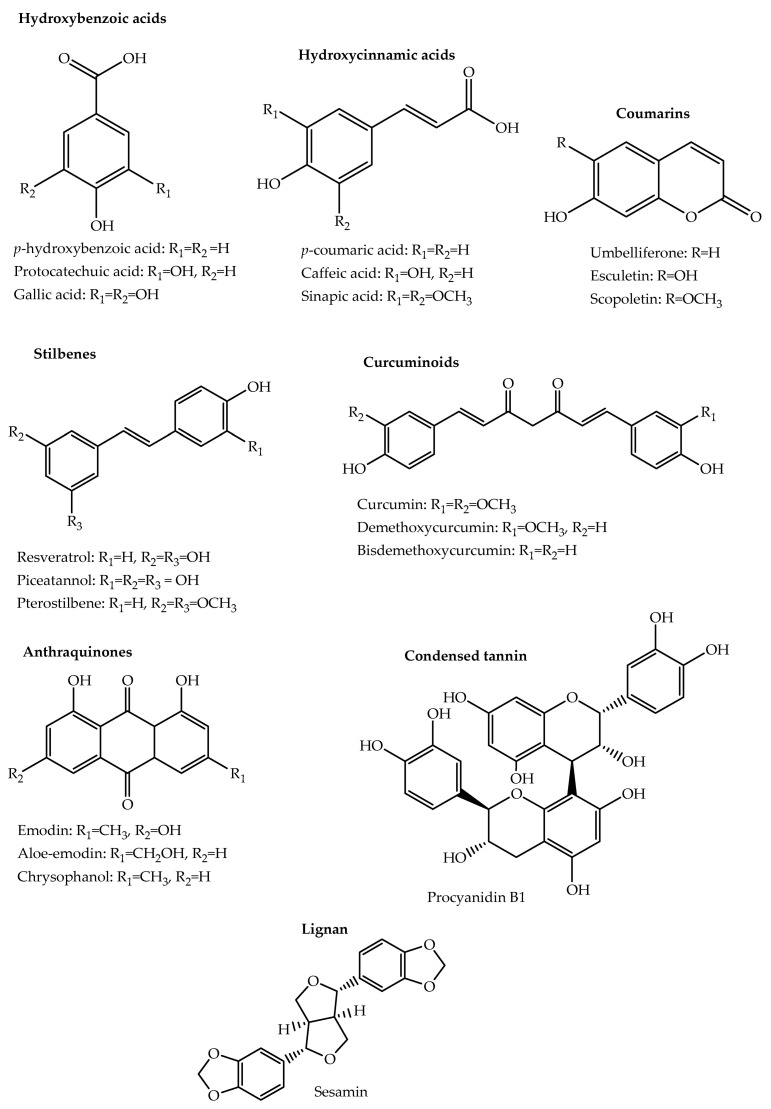
Structures of selected examples of phenolic acids, coumarins, stilbenes, curcuminoids, quinones, tannins and lignans.

**Figure 2:**
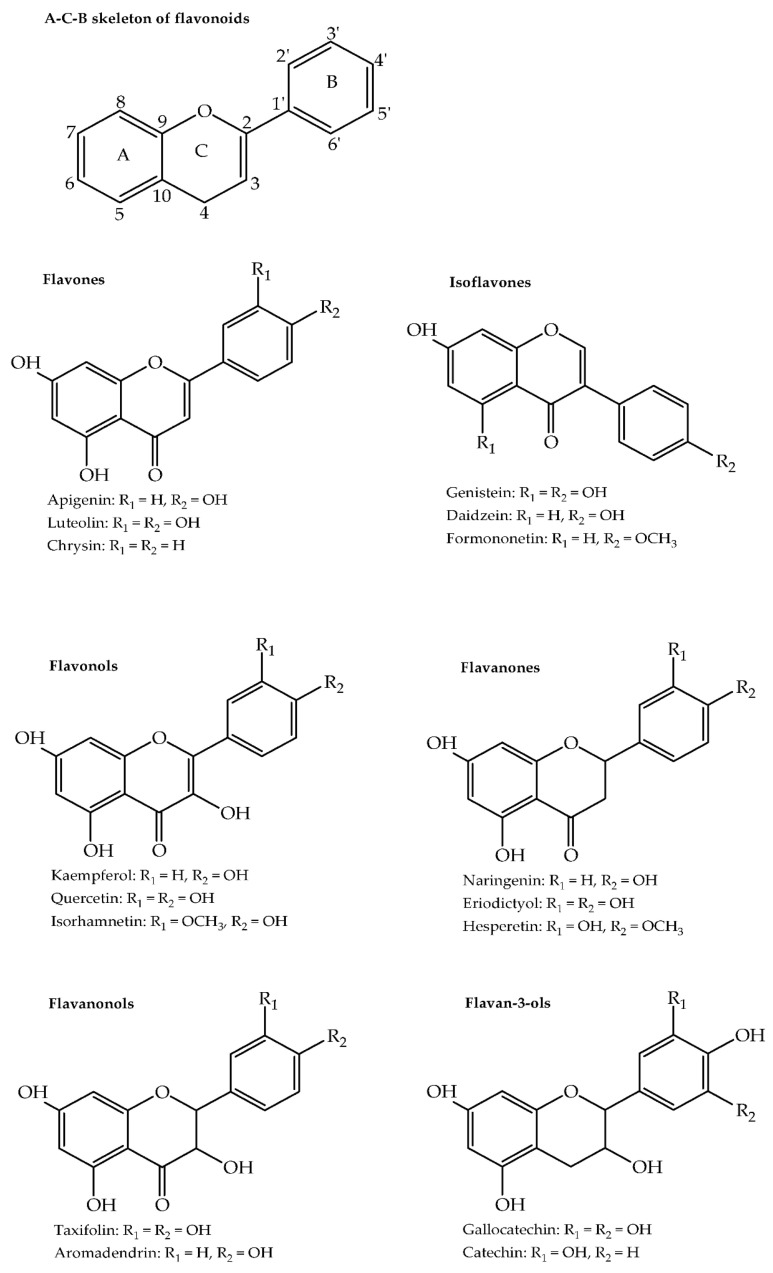
Structures of flavonoids.

The basic structure of phenolic compounds is characterised by the presence of at least one hydroxyl group directly attached to a phenyl ring. Based on the basic structural skeleton, they can be distinguished into phenolic acids, flavonoids, tannins, coumarins, lignans, quinones, stilbenes and curcuminoids. They are mainly synthesized through the shikimic acid and phenylpropanoid pathways [9,24]. Biosynthesis of the various phenolic compound classes is covered in several reviews [24,25,26]. Table 1 shows selected examples of phenolic compounds from the different classes that have been isolated from medicinal and aromatic plants. Structures of selected compounds from each phenolic class are shown in Figure 1 and Figure 2.

### 2.1. Phenolic Acids and Their Derivatives

Phenolic acids contain a carboxylic acid group in addition to the basic phenolic structure and are mainly divided into the hydroxybenzoic and hydroxycinnamic acids. Hydroxybenzoic acids are based on a C6-C1 skeleton and are often found bound to small organic acids, glycosyl moieties, or cell structural components. Common hydroxybenzoic acids include gallic, syringic, protocatechuic, *p*-hydroxybenzoic, vanillic, gentistic, and salicylic acids [9,24]. Gallic acid is present in cloves (*Eugenia caryophylata* Thunb.), while protocatechuic acid can be found in coriander (*Coriandrum sativum* L.), star anise (*Illicium verum* Hook. f.) and dill (*Anethum graveolens* L.). Hydroxybenzoic acid derivatives include syringealdehyde and vanillin, a major component in vanilla [24,28].

Hydroxycinnamic acids are based on a C6-C3 skeleton and are also often bound to other molecules like quinic acid and glucose. The main hydroxycinnamic acids are caffeic, *p*-coumaric, ferulic and sinapic acids. Caffeic acid is found among others in parsley (*Petroselinum crispum* L.), sage (*Salvia officinalis* L.) and ginger (*Zingiber officinale* Rosc.). *p*-coumaric acid is found in oregano (*Origanum vulgare* L.), thyme (*Thymus vulgaris* L.), basil (*Ocimum basilicum* L.) and many others. Derivatives of hydroxycinnamic acids include rosmarinic acid, an ester of caffeic acid; caftaric acid, an ester of caffeic and tartaric acid and chlorogenic acid which is an ester of caffeic acid and quinic acid [9,24,28].

### 2.2. Stilbenes

Stilbenes are based on 1,2-diphenylethylene, which has a C6-C2-C6 skeleton. They are often hydroxylated (stilbenoids) and can be found as aglycones, monomers, oligomers or glycosylated derivatives [44]. They can also be methylated or prenylated among other modifications [26]. *Trans*-resveratrol is the most common stilbene and is a precursor for many larger stilbenes. Medicinal plants belonging to *Eucalyptus* and *Pinus* genera are common sources of stilbenes [9].

### 2.3. Coumarins

Coumarins are benzopyrone derivatives occurring in all plant parts but mainly found in the flowers of plants belonging to several families. They may occur in a free or glycosylated state and are divided into six categories, namely simple coumarins such as coumarin and esculetin; furanocoumarins such as psoralen and imperatorin; dihydrofuranocoumarins such as anthogenol and felamidin; pyranocoumarins such as grandivittin and pseudocordatolide C; phenylcoumarins such as isodispar B and disparinol D; and bicoumarins such as dicoumarol [45,46]. They have a wide presence in medicinal and aromatic plants with various plant families having several hundreds of coumarins identified in each of them. Matos et al. (2015) summarise selected coumarins that have been identified in medicinal and aromatic plants belonging to several plant families, including *Apiaceae*, *Rutaceae*, *Asteraceae*, *Fabaceae* and *Thymelaeaceae* [46].

### 2.4. Lignans

Lignans consist of two phenylpropane units joined together by a *β*-*β*’ bond. They are divided into eight categories, namely dibenzylbutyrolactols, dibenzocyclooctadienes, dibenzylbutyrolactones, dibenzylbutanes, arylnaphthalenes, aryltetralins, furans and furofurans [25,47]. Phytochemical screening of 35 medicinal plants from Kenya revealed the presence of lignans in 68.5% of the plants with a very strong presence in leaves of *Phyllanthus sapialis*, roots of *Cyphostemma serpens* (A. Rich), the stem bark of *Zanthoxylum gilletii* (De Wild.) P.G. Waterman [48]. Several plants used in Asian traditional medicine, including *Schisandra glaucescens* Diels., *Schisandra* *chinensis* Baill., *Isodon* spp. and *Tripterygium* spp. are major sources of bioactive lignans [47].

### 2.5. Quinones

Quinones contain a di-one or di-ketone group and are distinguished into benzoquinones, naphthoquinones and anthraquinones based on their derivative molecules, benzene, naphthalene and anthracene. They may occur as monomers, dimers, trimers, glycosides or in reduced forms. In addition to the shikimate pathway, their biosynthesis also occurs through various other pathways, including the acetate and malonate pathway [49,50]. Eyong et al. (2013) presents a list of some of the different quinones that have been isolated from African medicinal plants [49].

### 2.6. Curcuminoids

Curcuminoids widely occur in *Curcuma* spp. especially in the rhizomes of *Curcuma longa* (turmeric) that has widespread use as both a medicinal and aromatic plant. There are three major curcuminoids, namely curcumin, demethoxycurcumin and bis-demethoxycurcumin of which curcumin is the major component and has been the most studied and shown to have various biological effects. The structure of curcumin consists of a keto-enol tautomeric unsaturated chain linking two aromatic rings bearing a hydroxyl and methoxy group. Demethoxycurcumin and bis-demethoxycurcumin are derivatives of curcumin in which one and both of the methoxy groups respectively are removed [51,52].

### 2.7. Flavonoids

Flavonoids form the largest group of natural phenolic compounds, and their structures are based on a 15-carbon phenyl benzopyran skeleton (C6-C3-C6, i.e., A-C-B rings) shown in Figure 2. Based on differences in the pyran ring, flavonoids can be categorised into flavones, isoflavones, flavonols, flavanones, flavanonols, flavan-3-ols and anthocyanidins. They occur in plants as aglycones and often the majority occur as glycosides except for flavan-3-ols, which are rarely glycosylated. Differing patterns of hydroxylation and methylation of the A and B rings consequently result in a variety of compounds for each flavonoid category [24,53].

Flavones have a double bond between C2 and C3, a keto function in C-4 and the B-ring is attached at C-2. The most common flavones in medicinal and aromatic plants are luteolin and apigenin, together with their glycosides. They are found in onion leaves, parsley, thyme, and many others. In isoflavones, the B-ring is attached at C-3 and the main isoflavones are daidzein, genistein and glycitein that are mainly found in leguminous plants. Flavonols are flavones bearing a hydroxyl group at C-3 and the major examples are kaempferol, quercetin and myricetin. In flavanones, the C-ring has no double bond between C2 and C3 and the major compounds in this category are naringenin, eriodictyol and hesperetin. Flavanonols, also called dihydroflavonols, for example, taxifolin and aromadendrin have the same saturated C-ring as flavanones but are hydroxylated at C-3. Flavan-3-ols, also referred to as flavanols, also contain a saturated C-ring but lack the keto group at C-4 and are hydroxylated at C-3. They can occur as monomers such as catechin and gallocatechin or as oligomers and polymers. In anthocyanidins, the C-ring lacks the keto group at C-4, is hydroxylated at C-3 and uniquely has two double bonds forming the flavylium cation. The common anthocyanidins are cyanidin, petunidin, pelargonidin, malvidin, peonidin and delphinidin and these largely occur as their glycosylated forms called anthocyanins [24].

### 2.8. Tannins

Tannins are high molecular weight polyphenolic compounds known for their ability to complex proteins and their astringent taste that deters insects, birds and herbivores from feeding on plants. They are found in most plant parts including the bark, wood, leaves, fruits and roots and can be synthesized as a defensive mechanism in response to pathogen attack and abiotic stresses such as UV radiation. *Caesalpinia spinosa*, *Salix caprea* and several medicinal plant species belonging to the genera *Acacia*, *Terminalia*, *Eucalyptus*, *Pinus*, *Betula* and *Quercus* are abundant sources of tannins [54,55]. Over 82 different tannins have been identified from different parts of several *Terminalia* spp. plants [56].

Based on their structures, tannins in plants can be classified into mainly the hydrolysable tannins and the condensed tannins, also known as proanthocyanidins. Hydrolysable tannins are built based on gallic acid and are divided into the gallotannins and ellagitannins. Gallotannins, e.g., pentagalloylglucose are formed when carbohydrates commonly glucose, or other polyols are esterified with gallic acid. In ellagitannins, e.g., ellagic acid, hexahydrodiphenic acid, which is a product of the oxidation of two gallic acid molecules is the basic structure.

A related group of tannins called complex tannins, e.g., eugenigrandin A has been defined as one in which either a gallotannin or an ellagitannin is attached to a flavan-3-ol through a C-C bond. Condensed tannins, e.g., procyanidins are formed by linking several flavan-3-ols like catechin and gallocatechin through C-C bonds to form polymers of varying sizes [9,54,55,56].

## 3. Extraction

The profile of phenolic compounds obtained from a particular medicinal or aromatic plant is heavily dependent on the chosen sample preparation and extraction techniques [57,58]. Prior to extraction, samples are often either freeze-dried, air-dried, or oven-dried. Before freeze-drying, samples are often initially snap-frozen in liquid nitrogen, which stops enzyme activity and metabolic processes and prevents further decompartmentalization of the plant tissues [59]. Dry samples are then stored in a freezer commonly maintained at about −80 °C. Effective sample drying can enable long-term storage of samples while at the same time preventing microbial growth and minimising loss of bioactive compounds [59,60]. The different drying methods may preserve varying levels of phenolic compounds. For instance, freeze-dried spearmint, *Mentha viridis*, showed the highest content of phenolic compounds followed by air-dried spearmint, whereas oven-dried samples gave the lowest content [60]. To maximize the extraction efficiency, after drying, samples are usually milled and homogenized to a fine particle size.

Phenolic compounds in the samples are usually extracted with solvents of varying polarities, including water, methanol, ethanol, acetone, ethyl acetate or alcohol-water and acetone-water mixtures depending on the compounds of interest. The choice of solvent is particularly important because depending on the chemistry of the phenolic compounds, they will have different solubility in the different solvents and thus, the nature of compounds and the yield obtained will differ with solvent type [57]. For instance, samples from seeds of *C. sativum*, *Nigella sativa* and *Trigonella foenum-graecum* extracted with 70% *v/v* methanol contained a higher quantity of phenolic compounds compared to those extracted with water [61]. The water extract of *T. vulgaris* gave a higher content of phenolics and flavonoids than the ethanol extract [29], and the methanol extract of *Cymbopogon citratus* (DC) Stapf. had a superior phenolic content than the ethanolic extract [62]. Rebey et al. (2012) found that 80% *v/v* acetone was the best extraction solvent for maximising content of phenolic compounds from *Cuminum cyminum* seeds [63].

The content of phenolic compounds extracted may also depend on the environmental conditions where the extracted plant material was grown including soil composition, light, rainfall and temperature. For instance, samples from different parts of *Capparis decidua* and *Capparis spinosa* harvested in a rainy month showed higher contents of phenolic compounds and flavonoids compared to those harvested in a drier month with much less rainfall [64]. *C. cyminum* seeds of Tunisian origin contained a higher quantity of phenolic compounds than those of Indian origin [63]. Generally, the determined levels of phenolic compounds will vary even between plants of the same species due to differences in various aspects, including maturity, developmental stage of the plant, analyzed plant tissue type, cultivar and pre-harvest factors.

Moreover, the extraction yields will also depend on the extraction time, temperature, sample to solvent ratios, number of repeat extractions, solvent purity and solvent pH. Increased extraction times and temperatures may increase the solubility of the phenolics in the extraction solvent implying a higher yield of compounds, but they may also increase the oxidation of the compounds and instead decrease the extraction yield. For extraction of phenolic compounds that are found bound to carbohydrates or proteins, acid, alkaline or enzyme hydrolysis can be done to release the compounds [57,58]. Oreopoulou et al. (2019) reviewed the effect of the various extraction parameters, including the extraction solvent, pH, temperature, extraction time, solvent-to-solid ratio and sample [65].

The traditional methods for extraction of phenolic compounds from medicinal and aromatic plants are maceration, Soxhlet extraction and heated reflux extractions. However, despite their relatively low cost and simplicity, they require long extraction times and large volumes of solvent [66,67]. Consequently, faster methods that use much smaller volumes of solvent are increasingly becoming popular. These advanced alternative methods include Ultrasound-Assisted Extraction (UAE), Microwave-Assisted Extraction (MAE), Accelerated Solvent Extraction (ASE), Sub-critical Water Extraction (SWE), Supercritical Fluid Extraction (SFE), and High Hydrostatic Pressure Extraction (HHPE). The use of these methods in extraction of phenolic compounds from plant material has been extensively reviewed in several papers and book chapters [58,68]. In particular, Oreopoulou et al. (2019) [65], Azwanida (2015) [67] and Arceusz et al. (2013) [66] reviewed the application of these methods in extraction of phenolic compounds from medicinal and aromatic plants.

UAE uses ultrasonic vibrations in either static or dynamic mode to increase penetration of the solvent into the sample during sonication of the mixture under controlled temperature and defined time. This makes it a simple yet fast and inexpensive extraction method [58,66]. MAE on the other hand uses microwave radiation to extract compounds from samples in solvents or solvent mixtures of high dielectric constants [65]. In ASE, also referred to as pressurized liquid extraction (PLE), common organic and aqueous solvents at elevated temperatures (typically 50–200 °C) and pressures (typically 4–20 MPa) are used for the extraction of phenolic compounds. The high temperatures and pressures improve the extraction kinetics and facilitate a more effective extraction in a short time. Subcritical water extraction (SWE) is an ASE extraction in which water is used as the solvent, and this process is particularly important in the extraction of compounds that have low or medium water solubility at room temperature [65,66]. SFE uses a supercritical fluid, most often carbon dioxide, as the extraction solvent. However, due to the low polarity of carbon dioxide, supercritical carbon dioxide extraction is not best suited for extracting phenolic compounds as these are largely polar. However, this problem can be counteracted by adding polar co-solvents such as methanol, ethanol, and acetone [58,67,69]. HHPE uses super-high hydraulic pressure typically in the ranges of 100 to 800 MPa to enhance mass transfer of components from the interior to the exterior of cell membranes leading to high extraction yields in a very short time. Since HHPE is usually done under ambient conditions, its useful for the extraction of thermo-sensitive compounds [58,65].

Sequential extraction with solvents of increasing polarity can also be done conventionally or using these advanced methods to extract different phenolic compound classes consecutively from the same starting plant material [70]. This is particularly important in cases where these extracts are being made for bioactivity tests as the different phytochemical composition of the sequentially obtained extracts may most likely imply different bioactivities [71]. Most studies on biostimulants and bioprotectant activity of plants have been conducted on their crude extracts made conventionally with a single solvent and only a few have considered sequential extraction with solvents of different polarities. However, sequential extraction would give the opportunity to test compounds from different phenolic classes that would otherwise be lost (not extracted) in a conventional one-step extraction with one solvent, hence not giving a comprehensive assessment of the bioactivity and efficacy of the plant in question.

**Figure 3:**
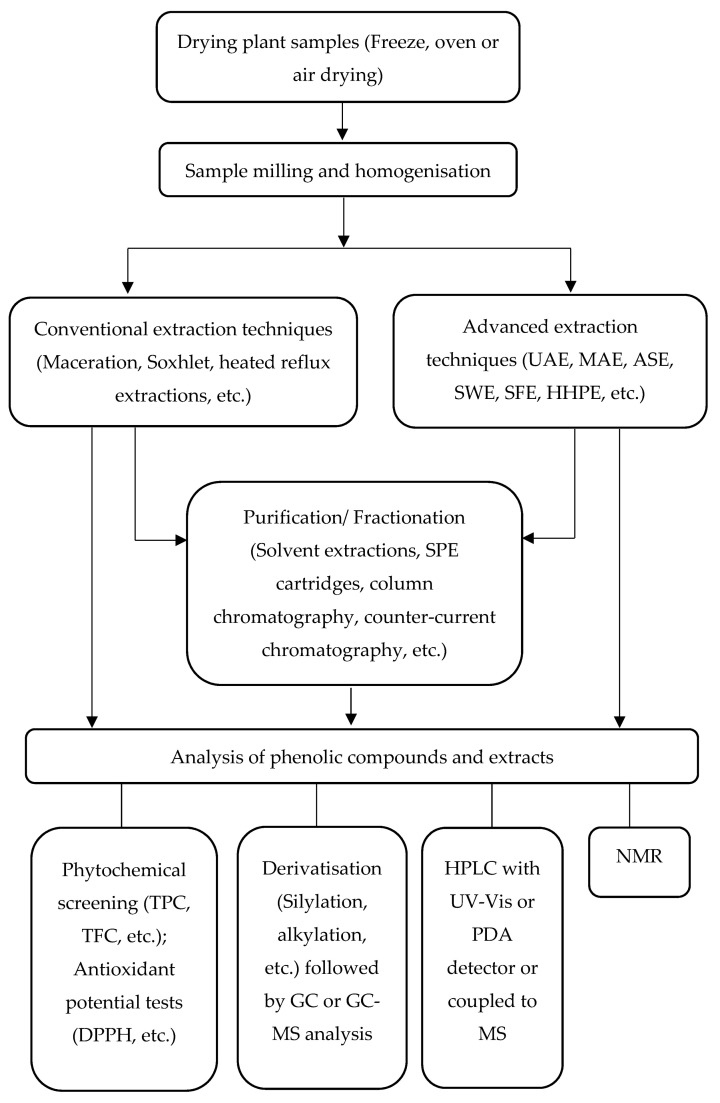
Summary of the characterisation process of phenolic compounds and extracts (UAE―Ultrasound-Assisted Extraction, MAE―Microwave-Assisted Extraction, ASE―Accelerated Solvent Extraction, SWE―Sub-critical Water Extraction, SFE―Supercritical Fluid Extraction, HHPE—High Hydrostatic Pressure Extraction, SPE―Solid Phase Extraction, TPC—Total Phenolic Content, TFC―Total Flavonoid Content, DPPH―2,2-diphenyl-1-picrylhydrazyl (DPPH), GC―Gas Chromatography, MS―Mass Spectrometry, HPLC―High-Performance Liquid Chromatography, UV-Vis—Ultraviolet-Visible, PDA―Photodiode array, NMR―Nuclear Magnetic Resonance).

In most cases, extraction is usually followed by purification or fractionation steps to separate the phenolic compounds from other compounds in the extract like carbohydrates and lipids. This can be done by employing solvent extractions, solid-phase extraction cartridges, column chromatography or counter-current chromatography [57]. The schematic diagram in Figure 3 summarises the different steps in the characterisation of phenolic compounds and extracts, from the preparation of plant material through extraction, purification, and subsequent analysis.

## 4. Phytochemical Screening

Plants are abundant sources of phenolic compounds. Preliminary phytochemical screening can give an indication of the phenolic constituents as well as their quantity in the assayed plant extracts. Simple qualitative tests based on colour observations or the formation of precipitates with specific reagents can show the presence of certain phenolic compounds. For instance, the formation of a green colour upon addition of 5% ferric chloride to a plant extract would imply the presence of phenols, whereas the formation of an intense yellow colour with sodium hydroxide solution that becomes colourless on addition of dilute hydrochloric acid would indicate the presence of flavonoids [72,73]. Quantitative tests often based on spectrophotometry can, on the other hand, give an impression of the amount of the phenolic compounds present in the extract. Commonly, these tests are used to determine the total phenolic content (TPC) or the content of particular phenolic types mainly the total flavonoid content (TFC) and seldom the total condensed tannins.

The TPC is often determined using the Folin–Ciocalteau method with varying modifications using gallic acid as a standard and hence the results are consequently expressed as gallic acid equivalents (GAE) per dry weight, for instance, mg GAE/g. TFC is mainly determined using a colorimetric method in which aluminium chloride forms complexes with flavonoids that can be measured spectrophotometrically. Often, a quercetin or catechin standard curve is used for the quantification and hence the results are expressed as quercetin or catechin equivalents (QE or CE) per dry weight for instance mg QE/g or mg CE/g. Total condensed tannins are commonly determined by the Vanillin assay with catechin as standard or the dimethylaminocinnamaldehyde assay [57,58,74]. The TPC and TFC values of selected medicinal and aromatic plants from different studies are shown in Table 2.

However, despite their simplicity, these “total” methods are inaccurate and only give a rough estimate of total composition as they involve interferences from other oxidants in the extract, external factors and formation of side products [57]. The Folin–Ciocalteau reagent, for instance, is not only unspecific but also does not detect all the phenolic types. It reacts with sugars, aromatic amines and ascorbic acid in the extract in addition to the phenolic compounds hence overestimating the determined values of the total phenolic content [58,74].

Tests on the antioxidant potential of the extracts can also be used as a proxy indication of the phenolic content. These tests include the 2,2-diphenyl-1-picrylhydrazyl (DPPH), ferric reducing antioxidant power (FRAP), and Trolox Equivalent Antioxidant Capacity (TEAC) assays, among others. Several studies have shown a positive correlation between the measured antioxidant activity and the phenolic content [61,75,76]. However, since the test is dependent on the phenolic content, values of the antioxidant activity are also dependent on the extraction solvent, extraction time, extraction method as well as the other factors cited earlier that affect the phenolic content determined from a given plant sample [61].

## 5. Analytical Methods

### 5.1. GC, HPLC and Their Hyphenation with MS

Separation, quantification and structural characterisation of phenolic compounds are mainly done using chromatographic and spectrometric methods. The chromatographic methods include gas chromatography (GC) and high-performance liquid chromatography (HPLC), whereas the spectrometric method is often mass spectrometry (MS). There are many reviews on the application of these methods in the analysis of phenolic compounds [57,58,66,68,74,79,80] and thus we shall only present a brief overview here.

Gas chromatography is a highly sensitive method suitable for analysis of volatile compounds. Phenolic compounds have very low volatilities and their analysis with GC requires that they are derivatised into more volatile forms often by alkylation or silylation of the hydroxyl groups with appropriate reagents [79]. The flame ionisation detector has been most commonly used in the past, but in recent years, most GC analyses have adopted mass spectrometers as detectors because the hyphenated method, GC-MS, presents better sensitivity and selectivity. For instance, various phenolic acids and flavonoids were identified from the aromatic plants *Origanum dictamnus,* *Eucalyptus globulus*, *O*. *vulgare*, *Mellisa officinalis* and *Sideritis cretica* by GC-MS after silylation of their extracts with *N,O*-bis(trimethylsilyl)trifluoroacetamide and trimethylchlorosilane [81]. Despite the possibility to analyse phenolic compounds by GC-MS either as aglycones or glycosides, analysis of whole high molecular mass polyphenolic glycosides is better suited for HPLC [74,79].

HPLC can analyse phenolic compounds and their derivatives at the same time, making it arguably the most suitable and most popular method for isolating, identifying and quantifying these compounds. HPLC analysis of phenolic compounds is usually done in reverse phase by employing C18 columns as stationary phase and a polar solvent system as mobile phase. The mobile phases are mainly based on methanol, acetonitrile and water acidified with very small proportions of either acetic, phosphoric or formic acid. Gradient elution tailored to the chemical nature and polarity of the compounds to be analyzed is preferred over isocratic elution since there is usually simultaneous analysis of phenolic compounds of different polarities. It is known that the more polar compounds have a preference for the mobile phase and elute earlier, whereas the less polar compounds will be more retained in the non-polar stationary phase and elute later [74,80]. Detection of phenolic compounds via HPLC is commonly done between 190 and 380 nm due to the presence of conjugated chromophores in their structures.

Various detection methods are employed of which the use of UV-Vis and diode array detectors (DAD or photodiode array detector, PDA) is most prevalent. Diode array detectors are preferred over UV-Vis because they can measure the absorption spectra of the compounds present in the sample over a wide range of wavelengths as opposed to the UV-Vis detectors that measure absorptions at single wavelengths [66,68]. Consequently, diode array detection provides more information about the purity and structures of phenolic compounds, which simplifies the identification of individual component compounds even in intricate plant extracts [74]. Kouasssi et al. (2017) [62] used HPLC with a UV-Vis detector to analyse the phenolic compounds in *C. citratus* (DC) Stapf., whereas Marques et al. (2016) [82] used it to analyse and quantify phenolic compounds in *Malpighia emarginata* DC. bagasse. HPLC with diode array detection was used to identify phenolic acids in the phytotoxic extracts obtained from the leaves, stems and roots of *Peganum harmala* [22], to check the purity of the nematicidal compound 3,4-dihydroxybenzoic acid isolated from the bark of the medicinal plant *Terminalia nigrovenulosa* [83], to study the isoflavone content of aqueous, hydroalcoholic and alcoholic extracts of six species of *Medicago* spp. [41] and to identify and quantify the phenolic components of the nematicidal extracts derived from three *Echinacea* species [21]. Identification and quantification of unknown compounds by HPLC is done by comparing their chromatographic data with that of standard reference compounds run under the same conditions. For proper quantification, calibration curves using standards at appropriate concentrations need to be obtained [80].

Just as in GC, for higher sensitivity and selectivity, HPLC is often coupled to MS. HPLC-MS (or simply LC-MS) is particularly useful in the identification and confirmation of structures and has found increased use in structural elucidation of phenolic compounds in recent years. Here, the phenolic compounds in the sample are separated on the HPLC before being passed onto the MS that also acts as another detector. The MS ionises and fragments compounds and separates the ions according to their mass to charge ratios giving information that can be used to elucidate structures of the compounds based on the fragmentation patterns. Electrospray ionisation (ESI) is the most common mode of ionisation used in LC-MS [80]. Benayad et al. (2014) used HPLC coupled to both ESI/MS and DAD to separate and characterise various phenolic compounds, including flavonoid glycosides from the seeds of fenugreek, *T. foenum-graecum* [84]. Amessis-Ouchemoukh et al. (2014) used UHPLC coupled to a microTOF-Q mass spectrometer equipped with an ESI interface to characterise compounds from the methanol extract of *Marrubium vulgare* leaves [85]. A similar system was used by Oldoni et al. (2019) to characterise the hydroalcoholic extract of *Moringa Oleifera* leaves which was found to predominantly contain flavonoid derivatives [86]. However, in most cases, without a standard, identities of compounds assigned from HPLC-MS can only be tentative. Certain identification can be achieved by additional analysis of these compounds by NMR.

### 5.2. Nuclear Magnetic Resonance Spectroscopy, NMR

NMR is the most reliable analytical technique for achieving complete structure elucidation of compounds, which is particularly important in the identification of new compounds. With this method, the entire structural framework of a compound, including how and where the different atoms may be attached can be identified with certainty. For instance, for an acylated and glycosylated flavonoid, NMR can be used to identify the exact nature of the aglycone, acyl group and glycosyl group. Additionally, we can identify the point of attachment of the sugar to the aglycone and where on the sugar the acyl group is attached [87].

Structure elucidation is done through a combination of one-dimensional (1D) and two-dimensional (2D) experiments. 1D experiments exist for analysis of both hydrogens and carbon but 1D ^1^H spectra are more frequently used. In a 1D ^1^H NMR spectrum, the most important parameters used in structure elucidation are the chemical shifts, signal multiplicities and coupling constants. The signal integral can also be used for the quantification of compounds. On the other hand, 2D experiments combine the information from the 1D parameters together with the observed cross-peaks to facilitate the complete identification of the structures of compounds [87,88]. These 2D experiments can be homonuclear where correlations between the same nuclei are observed or heteronuclear where the observed correlations are between different nuclei. Commonly, in the analysis of phenolic compounds, the homonuclear experiments include the Correlation Spectroscopy (COSY), Total Correlation Spectroscopy (TOCSY) and Nuclear Overhauser Enhancement Spectroscopy (NOESY) experiments which are all 2D ^1^H-^1^H experiments. The heteronuclear experiments involve 2D ^1^H-^13^C correlations and the two most popular experiments are the Heteronuclear Multiple Bond Correlation (HMBC) and Heteronuclear Single Quantum Coherence (HSQC).

#### 5.2.1. 1D ^1^H NMR

Phenolic compounds have diverse structures that can involve varying numbers of substituents. Every hydrogen atom that exists in a unique chemical environment in the structure will show a signal in the 1D ^1^H NMR spectrum at a specific chemical shift value usually between 0 and 12 ppm. The multiplicity of this signal gives an indication of the number of hydrogens on the nearest carbon atoms in the structure. The coupling constant measured in Hertz (Hz) gives information about the distance and dihedral angles between the coupled hydrogens. Thus, together, the signal multiplicity and the coupling constant of the multiplet will give information on the relative positions of the different hydrogens in the compound [88,89].

In phenolic natural products, examples of the usefulness of 1D ^1^H NMR include the case of double bonds, e.g., those in hydroxycinnamic acid derivatives where coupling constants distinguish whether the olefinic hydrogens are arranged in a *cis* or *trans* configuration [90]. *Trans* configurations, for instance, show larger coupling constants compared to the *cis* configurations. Additionally, coupling constants can also be used to determine the anomeric configuration of the sugar moiety and to distinguish similar sugars, especially the case of whether it’s glucose or galactose present in a compound. For instance, the anomeric hydrogen of a *β*-anomer shows a larger coupling constant of about 7–10 Hz compared to 1–4 Hz for an *α*-anomer [27,89,91]. One other important parameter in the 1D ^1^H NMR spectrum is the integration value of the signals which is equivalent to the number of hydrogens giving rise to the signal. This integral area of the signal can also be used to quantify the concentration of the assessed compounds [88,89].

Despite being the most used NMR technique, 1D ^1^H NMR has some limitations. Perhaps, the most common problem is the signal overlap observed in the spectra, especially in cases where there are multiple compounds in the same spectrum or commonly when analyzing a compound with multiple sugars. In 1D ^1^H NMR spectra of phenolic compounds containing one sugar molecule, it is easy to observe all the sugar ^1^H signals clearly in the spectrum. However, for compounds containing multiple sugars, there is often considerable overlap of the sugar signals especially in the non-anomeric sugar region and assigning chemical shifts to the different hydrogens of the different sugars will normally require the intervention of 2D experiments like the COSY, TOCSY and NOESY.

#### 5.2.2. 2D ^1^H-^1^H COSY

The COSY experiment shows cross-peaks representing correlations between hydrogens that are spin-coupled to each other in a structure. These cross-peaks may be seen for ^2^*J*_HH_, ^3^*J*_HH_ and even ^4^*J*_HH_, corresponding to couplings between hydrogens that are two, three or four bonds from each other. In a double quantum filtered (DQF) COSY, signals for non-coupled hydrogens do not appear, which helps to reduce crowding in the spectrum. The COSY experiment is particularly useful in assigning chemical shifts to the hydrogens on sugars. In sugar-like glucose, all the hydrogens will couple to those on adjacent carbon atoms and it is thus possible to assign the chemical shifts to each subsequent hydrogen by sequentially following the cross-peak pattern. This of course is dependent on the spectrum not being crowded and the cross-peaks not lying too close to the diagonal. Otherwise, a TOCSY spectrum can be used in tandem with the COSY to try to solve this problem [87]. For instance, Rayyan et al. (2010) used the COSY and TOCSY experiments to assign all the chemical shifts of the sugar hydrogens of various flavone C-glycosides that they isolated from fenugreek, *T. foenum-graecum* L. [91].

The 2D ^1^H-^1^H COSY has been used together with other NMR experiments in several studies to elucidate structures of various new phenolic compounds from different medicinal and aromatic plants. For instance, Khalafallah et al. (2009) identified 5-hydroxytephroapollin F, a novel prenylated flavonoid from the aerial parts of the medicinal plant *Tephrosia apollinea* [92]. Zhao et al. (2007) characterized five new 8-(3,3-dimethylallyl)-substituted flavonoid glycosides, including four flavonol glycosides and a flavanonol glycoside from the aerial parts of the medicinal plant *Epimedium koreanum* [93]. Li et al. (2017) characterized two new flavonoids, officinoflavonoside A and officinoflavonoside B from the aerial parts of *R. officinalis* [94].

#### 5.2.3. 2D ^1^H-^1^H TOCSY

Like the COSY, the TOCSY spectrum shows hydrogen–hydrogen correlations but unlike the COSY, the hydrogens do not have to be necessarily *J*-coupled directly. The TOCSY spectrum shows correlations between signals belonging to the same spin system. This is particularly useful in the assignment of chemical shifts to sugar hydrogens of phenolic compounds that have two or more sugar units as these always have overlapping signals in the sugar region and it may not be possible to easily figure out all the ^1^H chemical shifts from just the 1D ^1^H NMR and COSY spectra. Each of the sugars consists of a discrete spin system and once one of the sugar hydrogens, mostly the anomeric has been observed, the correlations of other hydrogens on the sugar can be seen in line with it clearly [87,89].

#### 5.2.4. 2D ^1^H-^1^H NOESY

The NOESY experiment shows correlations between hydrogens that are adjacent to each other in space, but not necessarily linked through bonds. In glycosylated flavonoids, the NOESY is useful in determining the point of attachment of the sugar to the aglycone or how the different sugars may be connected to each other [27,87]. For instance, Manguro and Lemmen (2007) used NOESY correlations to confirm the connection pattern of the sugars in kaempferol 3-O-[α-rhamnosyl-(1→2)]-[α-rhamnosyl-(1→4)]-*β*-glucoside-7-O-α-rhamnoside isolated from the leaves of *M. oleifera* [27]. The experiment can also be used to identify the stereochemistry of double bonds and sites of conjugation [95]. Krzyzanowska-Kowalczyk et al. (2018) used NOESY correlations to confirm structures of the phenolic compounds pulmitric acid A and pulmitric acid B from the aerial parts of *Pulmonaria officinalis* [95]. Additionally, they used NOESY correlations to confirm an *E*-configuration for the double bond as well the point of conjugation with a rosmarinic acid molecule in the structure of pulmitric acid B.

#### 5.2.5. 2D ^1^H-^13^C HSQC

The HSQC shows cross-peaks between hydrogens and carbon atoms that are directly attached together. Therefore, the cross-peaks observed in the HSQC are as a result of ^1^*J*_CH_ couplings. It thus makes it a very important spectrum for assigning chemical shifts to carbon atoms with directly bonded hydrogens. Once the ^1^H chemical shifts are known, the chemical shifts of the carbon atoms can be determined from their ^1^*J*_CH_ cross-peak [89]. The HSQC has thus been widely used in the characterisation of phenolic compounds from medicinal and aromatic plants, including *R. officinalis* L. [94], *P. officinalis* [95], *M. officinalis* [96] and *Atriplex halimus* [97].

#### 5.2.6. 2D ^1^H-^13^C HMBC

Unlike the HSQC, the HMBC mainly shows cross-peaks resulting from long-range couplings between hydrogens and carbons in the same compound. Usually the strongest cross-peaks are observed from ^2^*J*_CH_ and ^3^*J*_CH_ couplings implying two or three bond couplings respectively. ^1^*J*_CH_ and ^4^*J*_CH_ couplings are also seldom found in some high-resolution HMBC spectra. These coupling patterns are useful in assigning the chemical shifts of quaternary carbon atoms [96,98]. Since couplings can also occur across oxygen atoms, the HMBC can also aid the identification of linkage points of sugars to the aglycone in glycosylated phenolic compounds or even the linkage point of acyl groups to sugars in compounds that are both glycosylated and acylated [87]. The HMBC has been extensively used in the characterisation of compounds from several medicinal and aromatic plants, including *E. koreanum* [93], *T. apollinea* [92], *T. foenum-graecum* L. [91] and *M. oleifera* [27].

## 6. Metabolomic Analysis versus Conventional Natural Product Analysis

Natural product chemistry has undoubtedly been used as a route to the discovery of new bioactive compounds from plants. Conventional natural product analysis usually begins with the extraction of compounds from the plant biomass using selected solvents and extraction methods. The bioactive compounds are then isolated and purified from the crude extracts through bioassay guided fractionation. The structures of these compounds can then be elucidated using HPLC, MS, NMR, or other available analytical methods. However, there are many compounds present in different concentrations that can be extracted from each plant’s biomass. Furthermore, metabolomes of different plants contain several similar compounds and it is largely established that plants within a family synthesize similar compounds [99]. Therefore, the conventional approach is often time-consuming as it is almost certain that in search of a novel compound, you will always come across many already known compounds. Moreover, isolation and purification steps may involve some degradation, structural alterations and loss of some compounds [100,101].

With metabolomic analysis, on the other hand, the analysis is done on the crude extracts with no need for prior fractionation of the individual compounds. It aims to achieve simultaneous analysis of all the metabolites present in the extract, including their identification and/or quantification. Metabolomic analysis of phenolic compounds mainly employs LC-MS and NMR. LC-MS is advantageous owing to its high sensitivity and use of small sample volumes [100]. Ultra-performance liquid chromatography with MS (UPLC-MS) is even more sensitive and can detect metabolites better than LC-MS [102]. NMR is particularly relevant because it facilitates structure elucidation and easy quantification of metabolites as the size of the signals in the spectra is proportional to the concentration of the metabolites. It is also a non-discriminatory, non-invasive and reproducible method that maintains the integrity of the sample [101,103]. Data acquired from LC-MS and NMR is analysed using computational and statistical methods including chemometrics and multivariate analysis to unravel patterns that can be used to identify significant novel or bioactive compounds. This is mainly through the untargeted metabolomics approach, which is used when seeking to identify novel compounds. Untargeted metabolomics involves high-throughput screening to simultaneously analyse all the compounds that are detected above the noise threshold [102]. Untargeted LC-MS metabolomics study of *Myrothamnus flabellifolia* plants collected from three geographical regions corresponding to low, moderate, and high rainfall showed significant variability in their phenolic compound profiles [104]. UPLC-MS metabolomics was used to assess the impact of three drying methods on the phytochemical profiles of *Allium sativum* and *Allium cepa*. Freeze drying followed by microwave drying retained more secondary metabolites in both plants compared to air drying. *A. cepa* was shown to contain higher levels of flavonoids than *A. sativum* with quercetin derivatives being more abundant in freeze-dried *A. cepa* samples compared to the air-dried and microwave-dried samples [105]. Targeted metabolomics on the other hand involves quantitative measurements of already defined compounds. A targeted UPLC-MS-based metabolomics approach aimed at studying the tissue-specific phenolic distribution of two medicinal plants, *Acanthopanax senticosus* (Rupr. Maxim.) Harms. and *Acanthopanax sessiliflorus* (Rupr. Maxim.) Seem. showed that the targeted phenolic compounds displayed clear tissue specific accumulation patterns. The compounds were most abundant in the roots and the C6-C3-C6 type compounds had the most abundance in both plants [106].

Metabolomics can be used to study plant responses to biotic and abiotic stresses. Metabolite profiling of a simple crude extract can be used to study qualitative and quantitative changes in plants grown in different environments or subjected to different biotic or abiotic stresses. This can be used to identify molecular biomarkers for the breeding of more resistant and higher-yielding crops [107]. In fact, a metabolomics analysis of abiotic stress-resistant and susceptible plant species can lead to identification of the metabolites responsible for the defence and these can be a benchmark for the development of bioprotectants. NMR-based metabolomics has been used to study the effect of environmental factors and cultural practices on crop growth, for chemotaxonomic analysis and to analyse plant ecotypes [103].

## 7. Potential for Use of Medicinal and Aromatic Plants as Biostimulants and Bioprotectants in Agriculture

Medicinal and aromatic plants are widely spread in different parts of the world where they can be found growing in the wild or being cultivated by farmers. Thus, the identification of efficacious extracts and compounds in these plants that can enhance agricultural production is of great importance. The use of water extracts of bioactive plants is, for instance, economically viable for farmers especially those living in poor communities since they would only need to source the plants and mix them with water which is inexpensive and does not require any specialist knowledge. However, the identification of prevalent phenolic compounds in these plants that have bioactive effects is also equally important as it opens a pathway to industrial development of novel crop biostimulant and bioprotectant formulations based on these compounds. Plant extracts contain high proportions of phenolic compounds and several studies have proven the biostimulant and bioprotectant potential of phenolic compounds and extracts. This bioactivity has also been suggested to be due to synergy between the different components of the extracts, still including the phenolic compounds. Therefore, the proceeding sections present the biostimulant and bioprotectant roles of phenolic compounds as well as extracts that have been reported in different studies.

### 7.1. Biostimulant Potential

Several studies have shown the biostimulant effect of phenolic compounds and plant extracts on seed germination, rooting, shooting, fruiting and yield, among other growth parameters when applied to seeds, leaves, entire plants, or to the soil. It has also been shown that some of these phenolic compounds and plant extracts can also have inhibitory effects on growth parameters especially when applied at high concentrations, and this can also be dependent on the mode of application [108,109]. The biostimulants are absorbed into plant tissue and thus their efficacy depends in part on the permeability of the tissues and the chemical structure of the biostimulant compounds [3]. The efficacy has also been shown to vary with concentration applied, frequency of application and time of application. However, generally, most studies have considered full crude extracts rather than individual phenolic compounds. Table 3 shows selected medicinal and aromatic plants whose extracts have shown biostimulant activity in various plants.

Various extracts have been shown to improve seed germination and emergence. Aqueous extracts of *A. sativum* and *Azadirachta indica* increased percentage germination in rice [115]. Water extracts of *Acacia gourmaensis* and *Eclipta alba* were investigated for their potential to stimulate seedling emergence and growth in laboratory trials and to improve grain yield in field trials in sorghum and pearl millet [110]. In both cereals, either extract improved seed emergence significantly with up to 80% of sorghum seeds treated with the extracts emerging as compared to 66% for the non-treated seeds. Both extracts also stimulated growth of seedlings with *A. gourmaensis* showing a more profound effect. Furthermore, results of their field trials indicated that both extracts increased the plant vigour and grain yield but only the *E. alba* extract gave a significant improvement in grain yield in comparison to the untreated seeds. For instance, with sorghum, whereas *A. gourmaensis* produced a grain yield increase of 208.3 kg/ha, *E. alba* produced a more significant increase 1027.1 kg/ha in comparison to the non-treated seeds.

Extracts can reinforce the effect of traditional fertilisers. Zida et al. (2018) further carried out field tests to study the difference between treating sorghum seeds with only the *E. alba* extract, a fertiliser only or a combination of both [117]. Their results indicated that applying the *E. alba* extract alone at a concentration of 2.5% *w/v* produced a mean yield increase of 21% in comparison to non-treated seeds, whereas fertiliser alone resulted in an increase of 23%. When the seeds were treated with both the extract and the fertiliser, a significant mean yield increase of 66% was observed indicating the possibility of a synergistic interaction between the extract and fertiliser. It is thus reasonable to assume that the extract was a biostimulant that facilitated uptake of the nutrients from the soil and fertiliser by the plant.

One very popular medicinal plant whose extracts have been widely tested for biostimulant properties is *M. oleifera*. It has been shown to improve growth and yield in cauliflower [76], plum trees [111] and rocket plants [18], among many other studies. Rana et al. (2019) applied a 25 mL foliar spray of water extracts of *M. oleifera* per plant at various durations and frequencies after transplanting cauliflower seedlings to test their effect on growth and yield [76]. They assessed growth parameters including plant height, number of leaves per plant, length, breadth and weight of the largest leaf, and fresh weight of the roots and stems. In comparison to the water control, all the applied treatments positively influenced all these assessed growth parameters of the cauliflower plants with the largest effect being observed in the application where the seedlings were sprayed two weeks after transplanting and after every two weeks thereafter. The same application produced the highest gross and marketable yields in comparison with the control. Generally, they observed that increasing the frequency of application of the extract during growth gave better yield. On the other hand, Thanaa et al. (2017) evaluated the effect of foliar application of 4%, 5% and 6% *M. oleifera* leaf extracts on the yield and fruit quality of the Hollywood plums across two seasons [111]. Extracts were applied progressively at full bloom, fruit setting, and two weeks after fruiting. All concentrations had a positive stimulant effect, but it was the 6% extract that showed the highest increase in yield, colour, weight and firmness of the plums.

Plausible mechanisms for achieving these effects suggested by the different studies have included promotion of phytohormones, modulation of enzymes and enhancement of beneficial metabolites. These can then influence plant metabolism, photosynthesis, nutrient absorption, nitrogen assimilation and other physiological processes in plants to stimulate growth and development [2].

#### 7.1.1. Regulation of Phytohormone Activity

Phenolic compounds can stimulate growth and development in plants by regulating phytohormone activity [18,118]. Phytohormones like auxins, cytokinins and gibberellins are known to promote growth, whereas others like abscisic acid are growth inhibitors. The application of phenolic compounds and extracts has been found to increase levels of the former and decrease levels of the latter [18].

De Klerk et al. (2011) studied the effect of various phenolic compounds at concentrations ranging from 3 to 3000 μM on the formation of adventitious roots in 1 mm apple stem slices on a rooting medium in presence of sub-optimal concentrations of indole-3-acetic acid (IAA) and *α*-naphthaleneacetic acid (NAA) [118]. In the presence of NAA, rooting was mainly inhibited by most of the tested phenolics. However, with IAA, the *ortho*-diphenols (catechol, caffeic acid and chlorogenic acid), methylated *ortho*-diphenols (ferulic acid and vanillin) and the triphenols (gallic acid, phloroglucinol, pyrogallol and tannic acid) significantly promoted the formation of adventitious roots from the stem slices with ferulic acid having the most profound effect, having increased the roots from 0.9 to 5.8. However, it was also observed that the monophenol salicylic acid inhibited rooting. Since the auxins were applied at sub-optimal concentrations and no rooting was observed in their absence irrespective of the concentration of the phenolics, there was clearly an interaction between the auxins and phenolics that promoted rooting. Generally, the findings of their study, including phenolics promoting rooting in IAA not NAA, reducing the optimal concentration of IAA and promoting rooting in the initial five days when auxins are also supposed to be promoting rooting, indicated that the phenolics protected the IAA. A further investigation on the effects of ferulic acid and phloroglucinol on the dose-response curve of IAA and time of application indicated that they both showed antioxidant activity by inhibiting the decarboxylation of the IAA and protecting the tissue from oxidative stress. Salicylic acid was shown to promote the decarboxylation of IAA, hence explaining its inhibitory effect on rooting [118].

The triphenol, phloroglucinol has shown good biostimulant potential in several studies. da Silva et al. (2013) made a comprehensive review on the use of phloroglucinol in plant tissue culture [119]. According to the review, phloroglucinol strongly promotes the development of somatic embryos into plantlets, improves shoot proliferation, and promotes root initiation and development either independently or in the presence of other plant growth regulators. However, the compound can also have inhibitory effects on growth especially at high concentrations.

Extracts have also been shown to regulate phytohormone activity. Abdalla (2013) applied leaf and twig extracts of *M. oleifera* at concentrations of 1%, 2% and 3% to rocket plants at 7 and 14 days after planting and found that the 2% leaf extract and the 3% twig extract produced the best biostimulant effect having caused the most significant enhancements in plant height, fresh herb weight and dry herb weight [18]. Hormonal analysis showed that the amounts of auxins, gibberellins and cytokinins were high in the treated plants compared to the control with the highest values observed in the 2% leaf extract and the 3% twig extract in agreement with the observed growth stimulation effects. Similarly, all applied extracts reduced the levels of abscisic acid below the ones observed in the control with the lowest levels still observed in the 2% leaf extract and the 3% twig extract.

Despite the above studies showing influence of phenolic compounds and extracts on phytohormone activity, there is still generally a lack of an in-depth understanding of their mode of action and further studies are needed to uncover the underlying mechanisms on a molecular level.

#### 7.1.2. Modulation of Enzymes

Phenolic compounds and extracts contribute to defence mechanisms in plants by modulating the activity of antioxidant enzymes. Enzymes like catalase, peroxidase and superoxide dismutase, together with antioxidant compounds like ascorbic acid and flavonoids detoxify reactive oxygen species whose accumulation in periods of stress can cause damage to cell structures. In the study by Abdalla (2013), the *M. oleifera* extract decreased the activity of peroxidase, superoxidase dismutase and catalase enzymes as well as the measured lipid peroxidation levels while at the same time increasing the levels of phenolic compounds and ascorbic acid in treated rocket plants compared to the control [18]. This implies that the extract promoted growth by increasing antioxidant compounds that prevent oxidative damage hence lowering the activity of the antioxidant enzymes. These antioxidant compounds are well known to modulate processes involved in plant growth and development. On the other hand, in addition to improving plant height, leaf area, stem diameter, fresh weight and dry weight, foliar application and fertigation of tomato seedlings with aqueous garlic, *A. sativum*, extracts increased the activity of peroxidase and superoxidase dismutase in response to lipid oxidation that was shown by increased amounts of malondialdehyde caused by high concentrations of the extract [109].

The fresh and dry weights of tomato callus tissue and potted tomato plants were increased by application of 10^−5^ M solutions of benzoquinone, naphthoquinone, and anthraquinone with the most significant increase of over 100% in callus tissue being observed with naphthoquinone [120]. In this study, naphthoquinone was found to suppress the activities of IAA oxidase, polyphenol oxidase and ascorbic acid oxidase in tomato callus tissues, mung bean cuttings and tomato plants, and this was suggested to be the possible mechanism for the growth stimulation effect. For instance, in the tomato plants, decreases of 88%, 47% and 45% were observed in the activity of IAA oxidase, polyphenol oxidase and ascorbic acid oxidase, respectively.

Growth attributes can also be stimulated by accelerating the activity of enzymes involved in driving biosynthetic processes in plants. Ertani et al. (2016) evaluated the effect of three phenol-containing vegetal extracts, including those from hawthorn (*Crataegus monogyna* Jacq.) on the growth of maize plants and effects on sugar and phenolic metabolism [19]. Chemical characterisation showed that the hawthorn extract contained 1140 mg/L of total phenolic acids, including gallic, chlorogenic, vanillic, caffeic, *p*-coumaric and *p*-hydroxybenzoic acids with the latter being the predominant phenolic acid. The extract caused an increase in the dry weight of both roots and leaves of the maize plants. Application of a 1 mL/L hawthorn extract increased the activity of Phenylalanine ammonia-lyase (PAL) 11-fold for treated plants in relation to the untreated plants and the activity of the enzyme was linearly related with the total phenol content of the leaves. PAL is implicated in the phenylpropanoid biosynthetic pathway, where it catalyses the first step in the pathway. In addition to the hawthorn extract showing IAA-like activity, this stimulation of the phenylpropanoid metabolism was suggested to be partly due to the presence of the phenolic acids in the extract.

Phenolic compounds have also been observed to stimulate plant growth by facilitating nitrogen assimilation through enhancing the activity of nitrogen fixing enzymes. Abd-Alla (1994) studied the effect of spraying shoots and irrigating plants with *p*-hydroxybenzoic acid, catechol and phloroglucinol independently at concentrations of 10, 100 and 1000 μM on the nodulation and nitrogen fixation in soybean inoculated with *Bradyrhizobium japonicum* [108]. The results showed that at 100 μM, all the applied phenolics increased the nodule number, plant dry weight as well as the total nitrogen content with the highest effects being observed in phloroglucinol. The irrigation method was found to be more effective compared to spraying. However, the highest concentration of the three phenolics (1000 µM) applied by either method was found to have an inhibitory effect as seen from the significant reductions in the observed nodule numbers, nodule fresh weight, plant dry weight and the nitrogen content. Under the same conditions, the NADH-dependent enzymes glutamate dehydrogenase and glutamate synthase that are responsible for assimilating excess ammonia showed increased activity. For instance, irrigation with 100 μM of phloroglucinol gave an activity of 27.8 and 19.3 for glutamate synthase in the treated and control plants, respectively. Because ammonia is the product of nitrogen fixation in the nodules, this implies that the phenols enhanced the uptake of nitrogen hence increasing the activity of these enzymes. This was also supported by the observed increase in the soluble protein in the nodules that showed a similar trend.

#### 7.1.3. Enhancement of Useful Metabolites

The biostimulant activity of the extracts and phenolic compounds has also been shown through demonstrating an increase in the quantities of useful metabolites implicated in growth and development when applied to seeds or seedlings. In addition to the stimulation of endogenous phenolics, some commonly stimulated metabolites include chlorophyll, carotenoids, proteins, sugars and amino acids among many others. Enhancement of antioxidant metabolites like endogenous phenolics and ascorbic acid helps plants to counteract the accumulation of reactive oxygen species, whereas an increase in chlorophyll helps plants to improve their photosynthetic rates. *M. oleifera* extracts increased the levels of chlorophylls, carotenoids, proteins and sugars in rocket plants. The stomatal conductance and photosynthetic rate of the plants were also improved [18]. Vegetal extracts of hawthorn increased levels of phenolic acids, chlorophyll as well as the protein, glucose and fructose in the roots and leaves of maize [19]. *T. vogelii* extract significantly increased the levels of chlorophyll and flavonoids in beans [112]. Naphthoquinone increased the levels of proteins and carbohydrates in tomato plants [120].

### 7.2. Bioprotectant Potential

Pathogens cause many diseases in plants and lead to postharvest spoilage of various crops. They cause mycotoxin accumulation in grains and rot in fruits. Insect pests can infest plants at different growth points causing damage to plant parts and affecting growth while nematodes infest roots causing root galls that lead to nitrogen deficiency and crop stunting. Phenolic compounds and extracts from medicinal and aromatic plants have been tested widely in laboratory and field tests for their antimicrobial, anti-insecticidal and nematicidal effects against these pathogens and pests in crops. Table 4 and Table 5 summarise the bioprotectant activity of selected phenolic compounds and extracts respectively.

#### 7.2.1. Antimicrobial Tests

Antimicrobial activity of phenolic compounds and extracts has been tested on various fungal and bacterial strains that cause common plant diseases in vitro [115,128,133]. Antimicrobial activity can be assessed by determining the diameters of zones of inhibition of growth of the pathogens after a determined incubation time in vitro or it can be expressed in terms of percentage inhibition of radial growth of the pathogen mycelia. The minimum inhibitory concentration (MIC) which is the lowest concentration of the tested extract or phenolic compound that inhibits the growth of the pathogen is often determined using methods such as agar well diffusion, disc diffusion and micro broth dilution on growth media.

Ahmed et al. (2002) tested the effects of 1:1, 1:2, 1:4 and 1:8 *w/v* extracts of *Polygonum hydropiper, A. cepa, A. sativum* and *A. indica* on the control of seed borne *Bipolaris oryzae* in rice [115]. Despite all extracts being effective, the 1:1 *w/v* extracts of each plant were most effective causing fungal reductions of 66.7 to 83.3% compared to the other concentrations. 1:1 *w/v* extracts of *A. sativum* and *A. indica* were found to be the most effective with extracts of the two causing percentage reductions in fungal infection of 91.7% and 83.3%, respectively, when the seeds were soaked in the extracts. The same extracts at the same concentration also showed the highest inhibition of mycelial growth of the pathogen but *A. indica* showed a higher percentage inhibition with 43.32% and 41.01% in the poison food and cup methods, respectively, compared to 36.36% and 38.76% for *A. sativum* in the poison food and cup methods, respectively. For all conducted tests, clearly the effect was concentration-dependent with effectiveness increasing with concentration. A similar observation was made by Jyotsna et al. (2017) who reported that 0.5% leaf extracts of ten different plants were significantly more effective in reducing the mycelial growth of the same pathogen than the 0.2% extracts [133].

Zida et al. (2008) used laboratory tests to examine the effect of water extracts of *A. gourmaensis* and *E. alba* on the control of seed-borne fungi in sorghum and pearl millet [110]. Both extracts showed significant antifungal effects against *Phoma sorghina* in both sorghum (27% and 72% reduction in incidence by *A. gourmaensis* and *E. alba*, respectively), and pearl millet (86.4% and 72% reduction in incidence by *A. gourmaensis* and *E. alba* respectively). Both extracts also reduced the incidence of *Curvularia lunata* and *Fusarium moniliforme* in pearl millet by percentages ranging from 52.1% to 86%. The *A. gourmaensis* extract was also able to reduce the incidence of *Colletotrichum graminicola* on sorghum seeds by 68.5%.

Some studies have compared the antimicrobial activity of plant extracts in vitro and in vivo. Aqueous leaf extracts of *A. indica*, *Calotropis procera*, *Lantana camara* L., and *O. basilicum* L. inhibited the in vitro mycelial growth of *Curvularia tuberculata* and two isolates of *Alternaria alternata* [131]. *L. camara* completely inhibited spore germination of *C. tuberculata*, whereas *O. basilicum* L. completely inhibited the germination of both isolates of *A. alternata*. In vivo tests of these two extracts on infected pear plants showed that *L. camara* protected the fruits from rot caused by *C. tuberculata* by 80%, whereas *O. basilicum* L. protected the fruits from rot caused by *A. alternata* by 82% and 85% for both isolates of the pathogen.

Individual phenolic compounds have also shown potent bioprotectant potential against pathogens in vitro. Chong et al. (2009) tested the antimicrobial activity and fungitoxicity of syringic acid, caffeic acid and 4-hydroxybenzoic acid against *Ganoderma boninense* that causes basal stem rot in oil palms [128]. By measuring the radial growth of the fungus on potato dextrose agar treated separately with the three phenolic acids at different concentrations between 0 and 2.5 mg/mL over 14 days, syringic acid was found to be the most fungitoxic to the pathogen. Even at the lowest tested concentration of 0.5 mg/L, syringic acid inhibited growth of the pathogen for up to five days and all the other higher concentrations caused total inhibition of radial growth of the pathogen. Only minor inhibitory effects were observed for caffeic and 4-hydroxybenzoic acid at 2.5 mg/mL.

The phenolic acids, *p*-coumaric acid, caffeic acid, ferulic acid, sinapic acid and gallic acid; the flavonoids, catechin, naringenin, quercetin and rutin; the stilbene resveratrol; as well as the compounds, coumarin and catechol were found to be effective against four strains of *Xylella fastidiosa*, a bacterial pathogen that causes disease in many plants, including grapes, almonds, citrus and coffee among others [123]. The MICs ranged between 100 and 2000 µM with catechol, caffeic acid and resveratrol showing the strongest activity against the tested strains as shown by their lowest MIC values of 100–200 µM.

Food pathogens and spoilage microorganisms are also well known to cause massive losses of crops, especially fruits and grains in storage, by causing rot or through mycotoxins that cause contamination. Chemicals are commonly used to control these, but their residues often accumulate in the food that poses a health risk to the human consumers. Many of these pathogens are also increasingly becoming resistant to chemical fungicides and bactericides and this has favoured interests for research into natural control substances from plants [62,122,129,130,134].

Several studies have shown the potential of plant extracts and phenolic compounds from medicinal and aromatic plants as effective antimicrobial agents against these food pathogens and storage microorganisms. Ozcan (1998) explored the inhibitory effects of 31 spice extracts from different plant parts on the growth of the mycotoxic fungus, *Aspergillus parasiticus* [129]. 2% extracts of black thyme (*Thymbra spicata* L.), oregano (*O. vulgare* L. ssp.), savory (*Satureja hortensis* L.) and wild thyme (*Thymus serpyllum* L.) in that order were found to be most effective causing complete inhibition of the fungus in vitro. At a concentration of 1.56 g/L, aqueous extracts of *Origanum compactum* inhibited radial growth of *Penicillium digitatum* mycelia by more than 97% inhibition within one day, with 100% inhibition being observed with 25 g/L extracts [130]. Methanolic and ethanolic leaf extracts of lemongrass, *C. citratus* (DC) Stapf. were effective in inhibiting the in vitro growth of *Fusarium graminearum* and *Fusarium oxysporum* sp. *tulipae* isolated from corn seeds [62]. The methanol extract was more effective compared to the ethanol extract showing MIC values of 25 μg/mL and 12.50 μg/mL for *F. graminearum* and *F. oxysporum* sp. *tulipae* respectively. The MIC values for the ethanol extract were double those of the methanol extract for each *Fusarium* strain. The higher inhibitory effect of the methanol extract was suggested to be due to its high total phenolic content of 118.14 mgGAE/g compared to just 35.43 mgGAE/g for the ethanol extract. This study’s evaluation of phenolic compound content showed that both extracts contained several phenolic acids and flavonoids, including *p*-coumaric acid, protocatechuic acid, quercetin, kaempferol and rutin.

Cetin-Karaca and Newman (2015) used the broth microdilution method to evaluate the antimicrobial potential of plant derived phenolic compounds, including chlorogenic acid, coumarin, curcumin, ellagic acid, eugenol, (-) epicatechin, rosmarinic acid, rutin, tannic acid, and xanthohumol against various strains of *Bacillus*, *Listeria* and *Clostridium* pathogens that are common food contaminating bacteria [122]. The growth inhibition of the studied bacteria varied significantly with the concentration and type of the applied phenolic compound as well as the species of the bacterial strain. With some compounds, the strains were observed to recover after 24 h and become resistant to the compound. Generally, xanthohumol and ellagic acid were among the most effective compounds, all with MIC values < 20 ppm.

Phenolic compounds also help to reduce postharvest stress in plants and to delay senescence. For instance, postharvest application of 2 mg/mL of salicylic acid to ‘Kensington Pride’ mangoes by dipping or vacuum infiltration significantly reduced the severity of anthracnose disease caused by the fungal pathogen *Colletotrichum gloeosporioides*, by delaying fruit skin ripening [124]. In addition, changes in the skin colour and firmness of the mangoes were also significantly slowed down, thereby increasing their shelf life by about 2 days. Salicylic acid and its derivative methyl salicylate are also known key signal molecules in the induction of systemic acquired resistance against disease in plants [135]. Phenolic compounds can thus have indirect bioprotectant activity through eliciting the induction of natural disease resistance in plants [12,135].

It has been suggested that the antimicrobial mechanism of plant extracts and phenolic compounds on the bacterial pathogens is through damaging the cell membrane of the bacteria by lowering the pH of the cytoplasm and causing hyperpolarisation of the membrane [134]. Scanning electron microscopy of *Bacillus subtilis* and *Listeria monocytogenes* treated with chlorogenic acid at MIC of 10 ppm showed some cells of the former to decrease in size while those of *L. monocytogenes* showed damaged cell walls with material adhering to the outside of cells and some cells were even empty. All these observations suggest that the cell walls and membrane were broken, and their contents were leaking [122].

Antibacterial activity of the phenolic compounds and plant extracts has been suggested to be related to the hydroxyphenyl structure and methylation. Polyphenolic compounds having pyrogallol groups in their structure were shown to have stronger antibacterial activity than those with catechol or resorcinol groups in their structures. Similarly, plant extracts whose polyphenolic constituents have pyrogallol groups show higher antibacterial activity [136,137]. Sinapic and ferulic acids which are methylated were found to be less effective than their non-methylated counterparts, *p*-coumaric, caffeic and gallic acids, against the bacterial pathogen *X. fastidiosa*. Though it could not be concluded that the activity of the acids against the pathogen was directly correlated to the number of hydroxyl groups in the structure, it was observed that hydroxyl groups positioned in the meta-position favoured antibacterial activity [123].

#### 7.2.2. Insecticidal Activity

Phenolic compounds show bioprotectant activity against insects that feed on plant produce through antifeedant mechanisms and by reducing their ability to produce abundant offspring [20,138,139]. Phenolic acid extracts of *Juglans regia* L. and *Mentha piperita* L. reduced the feeding ability of peach potato aphid, *Myzus persicae*, and the bird-cherry oat aphid, *Rhopalosiphum padi* L., on pea and winter wheat seedlings with deterrence indices of up to 42.5% [20]. These extracts additionally decreased the number of larvae produced by the mature aphids and further increased the time required for the produced larvae to develop. Whereas the aphids in the control experiment could produce up to 5.2 larvae a day, aphids in the experiments sprayed with 0.2% extracts had their reproductive capacity reduced up to a daily fecundity of just 1.02 larvae a day. The efficacy of both extracts in controlling the aphid numbers was 70% and over for *M. persicae* and over 80% for *R. padi* L. Tomczyk and Suszko (2011) demonstrated the role of phenolic compounds on mortality and reduction of fecundity in feeding insects by showing that extracts of *S. officinalis* in which phenolic compounds had been removed had significantly less toxicity than the full extracts against the two spotted spider mite, *Tetranychus urticae* Koch [138]. There was a lower percentage of dead eggs, larvae and adults as well as lower reduction in fecundity of surviving females in the extract without phenols implying that the phenols were necessary for the enhanced toxicity of the extract and possibly indicating that bioactivity is due to synergy between the phenolics and other compounds present in the extract.

Gallic acid, catechin, epigallocatechin gallate, *p*-coumaric acid, salicylic acid and quercetin isolated from the 70% acetone extract of acerola (*Malphigia emarginata*) bagasse increased larval mortality and length of the pre-pupal phase of fall army worm, *Spodoptera frugiperda* when added to the pest’s diet. LC_20_ and LC_30_ values of 648.18 and 1614.35 mg/L were reported [82]. Despite not causing mortality, the lignan, sesamin, isolated from *Piper mullesua* caused feeding deterrence and growth inhibition in 4^th^ instar larvae of *Spilarctia obliqua* with 50% feeding deterrence and 50% growth inhibition being caused by effective doses of 3856 ppm and 6212 ppm of sesamin, respectively [140].

Field experiments showed that aqueous extracts of *T. vogelii* and *M. oleifera* were effective in reducing populations of the adult insect pests *Phyllotreta cruciferae, Diabrotica undecimpunctata* and *Dacus cucurbitea* in water mellon with *M. oleifera* reducing *P. cruciferae* populations by up to 62% and *T. vogelii* reducing populations of *D. undecimpunctata* and *D. cucurbitea* by up to 64% [114]. The efficacy of the two extracts was dose-dependent and varied with the tested pests and time in the planting season, with lower populations of the pests being observed later in the season. The incidence of *P.* *cruciferae* and *D*. *undecimpunctata* was also reduced in cucumber by aqueous extracts of *A. indica* and *Zingiber officinale* [116]. Aqueous extracts of *T. vogelii at* 5% *w/v* also reduced the infestation of aphids on beans in the field [113]. The insecticidal activity of *T. vogelii* is due to the presence of known toxic phenolic compounds like the isoflavonoid rotenone. The vulnerability of different insects to the different plant extracts differs significantly, implying that they react to the contained phenolic compounds differently.

#### 7.2.3. Nematicidal Activity

Nematodes attack a wide variety of crops and cause huge yield losses. One of the most economically significant and thus widely used nematodes in studies of plant–nematode interactions are the *Meloidogyne* ssp. Their larvae infect plants forming root galls and causing nitrogen deficiency leading to the death of young plants and lowering of yield in mature plants [83]. Ohri and Pannu (2010) comprehensively reviewed the roles of different phenolic compound groups in plant–nematode interactions [141]. In vitro bioassays examining nematicidal activity often include testing the effect on chemotactic behavior as well as inhibition of motility and egg hatching [21,121,142].

Aqueous leaf extracts of *L. camara* immobilized 96% of the tested second stage juveniles of *Meloidogyne incognita* in 48 h [132]. Similarly, prepared extracts of the medicinal plants *Argemone Mexicana* and *Achyranthus aspera* caused 98.92% and 92.36% mortality of the *M. incognita* second stage juveniles in 72 h, respectively, and both extracts completely inhibited hatching [142].

Root extracts (1000 µg/mL) of *Echinacea angustifolia* and *Echinacea pallida* were more effective than extracts derived from the aerial parts in mortality and egg hatching bioassays of *M. incognita* second-stage juveniles [21]. Root extracts caused mortalities of 71.3% (LC_50_, 352 µg/mL) and 69.6% (LC_50_, 415 µg/mL) after 48 h for *E. angustifolia* and *E. pallida* respectively, whereas the aerial extracts caused 65.2% (LC_50_, 487 µg/mL) and 65.9% (LC_50_, 483 µg/mL) respectively. Echinacoside and other caffeic acid derivatives were identified as the main phenolic compounds in the extracts.

Aqueous extracts of *T. vulgaris* and *Punica gratum* caused 100% mortality of *M. incognita* nematodes in 72 h and 100% egg hatching inhibition in 30 days [143]. The same extracts also immobilized 71.4% and 95.7% of *Helicotylenchus dihystera* nematodes in 72 h. The study suggested that the inhibitive action of the extracts was in part due to these extracts inhibiting acetylcholinesterase activity. The results showed a concentration-dependent suppression of acetylcholinesterase activity in the nematodes with for instance the standard solution (25 g/500 mL) causing 98.7% suppression and half the standard solution concentration causing 46.7% suppression in *H. dihystera* nematodes.

Several phenolic compounds showed repellent activity against the nematodes *Radopholus similis* and *M. incognita* in bananas. In chemotaxis assays, the minimum concentrations that showed a repellent factor of 0.5 ranged between 24 and 257 µg/mL and were observed with the compounds *p*-coumaric acid and umbelliferone, respectively [121]. In the motility inhibition assays, salicylic acid caused death of both nematodes with LC_50_ values of 46 and 370 µg/mL for *M. incognita* and *R. similis* respectively in 72 h. The compound also completely inhibited motility in *M. incognita* while the highest motility inhibition of 85% in *R. similis* was caused by ferulic acid in 72 h. Generally, egg hatching was inhibited by flavonoids in *R. similis*, whereas only caffeic and salicylic acids had hatch inhibitive effects for *M. incognita*.

3,5-dihydroxybenzoic acid and gallic acid (0.5% *v/v*) isolated from aerial parts of *Rubus niveus* caused 100% and 94% mortality of *M. incognita* larvae, respectively after 48 h. The ethanolic extract of the same plant at the same concentration caused 52% mortality in the same time frame [144]. A direct contact bioassay by Nguyen et al. (2013) showed that at 1mg/mL, the other isomer 3,4-dihydroxybenzoic acid caused 85% hatch inhibition after three days of incubation and 94.2% mortality of second stage *M. incognita* juveniles after 12 h [83].

Several other phenolic compounds have caused mortalities in nematodes, including pyrogallol (99.4% mortality), ethylgallate (96.1%), gentistic acid (75.5%), juglone (100%), apigenin 7-O-glucoside (98.1%) and 7-hydroxycoumarin (90.3%) [126,127]. The high mortalities presented by these compounds suggested that vicinal trihydroxy, metahydroxy, quinone and coumarin groups seem to enhance nematicidal activity.

#### 7.2.4. Herbicidal Activity

Phenolic compounds and extracts can act as allellochemicals that can control harmful weeds that compete with plants. They show toxicity towards the weeds by inhibiting physiological processes such as photosynthesis. A review by Li et al. (2010) highlighted the various mechanisms by which allelopathic activity occurs in plants, including inhibition of cell division, elongation and plant nutrient uptake as well as affecting photosynthesis, respiration, enzyme function, synthesis of endogenous hormones and protein synthesis [145].

Growth and biomass characteristics, including leaf and root fresh weight, dry weight and length of *Dactylis glomerata* L. seedlings were decreased by applying *p*-hydroxybenzoic acid in concentration ranges of 0.1 to 1.5 mM [125]. The magnitudes of decrease were up to 43% for leaf fresh weight, 63% for leaf dry weight, 50% for root fresh weight, 56% for shoot length and 41% for root length. The phenolic also decreased the relative water content, leaf osmotic potential, photosynthetic efficiency, quantum yield of photosystem II photochemistry, leaf carbon and leaf protein in comparison to the controls. The decrease in these parameters suggests that the phytotoxic mechanism involves reducing nutrient uptake, inhibition of photosynthesis and causing biochemical stress.

Phenolics derived from different plant parts show different, concentration-dependent levels of phytotoxicity. The leaf powder of *P. harmala* amended into the soil showed greater growth inhibition of the weeds *Avena fatua* and *Convolvulus arvensis* than the stem and root powders in line with the leaf powder showing the highest concentration of total phenolics of the three [146]. Treatment with aqueous extracts of the three plant parts showed the same effect on the growth and germination of the seedlings with the inhibitory effect being in the order of leaves > stems > roots. For instance, 16% leaf, stem and root extracts of *P. harmala* contained 0.68, 0.54 and 0.25 mg/mL total phenols respectively and inhibited root length in *C. arvensis* seedlings by 64%, 50.7% and 38%, respectively [22]. HPLC screening showed the presence of gallic, vanillic, 3,4-dihydroxybenzoic, 4-hydroxybenzoic, caffeic, syringic and ferulic acids in the leaf extract. 4-hydroxybenzoic, syringic and ferulic acids were absent in the stem extract, whereas vanillic, 3,4-dihydroxybenzoic, caffeic and ferulic acids were absent in the root extract. The involvement of phenolics in the phytotoxic effect was shown when tests in which activated charcoal was added to the amended soils showed less growth inhibition than in those without. Activated charcoal binds the organic compounds, including the phenolics. Determination of the total phenol content showed large decreases in the determined values after addition of the activated charcoal [146].

## 8. Conclusions and Suggestions for Future Work

The research reviewed here shows the potential of phenolic compounds and extracts from medicinal and aromatic plants as biostimulants and bioprotectants in agriculture. Many studies have been carried out using uncharacterised whole plant extracts. It has been shown that these extracts are efficacious partly due to synergy between component compounds, including non-phenolics, but it is also important to understand which phenolic compounds potentially contribute to the bioactivity and to decipher how much of the supposed synergistic effect can be attributed to these metabolites. Studies that have tested individual phenolic compounds have largely considered simple phenolic compounds (low molecular weight secondary metabolites) and yet medium and higher molecular weight polyphenolic compounds are also abundant in plants and are also potentially bioactive. With developments in separation and analytical methods, many complex phenolic compounds be can efficiently isolated and unambiguously identified.

It is also important to note that majority of the tests have been done in vitro. However, laboratory and field conditions are totally different and effective concentrations in laboratory experiments may not necessarily translate to field conditions. There is therefore a need to understand the technicalities involved in scaling from laboratory to field experiments after finding in vitro activity in either individual phenolic compounds or whole plant extracts. As well, looking at the studies cited in this review, there is a big disparity in the concentrations tested in the different studies and this makes it hard to cross compare results from different species. However, given that one of the major challenges in natural product chemistry is a low abundance of individual entities, efficacy of these compounds and extracts at low concentration titres would be important.

Despite various attempts, more investigations into the mechanisms of how these compounds and extracts bring about biostimulant and bioprotectant effects are needed. Phenolic compounds and extracts may not have direct effects on growth parameters or direct bioprotectant effect against pests and pathogens, but rather indirect effects, for instance on the soil and plant microbiome, or by acting as elicitors of induced disease resistance, that may subsequently facilitate the biostimulant or bioprotectant effects. There is currently little research into the indirect effects of exogenously applied plant extracts and phenolic compounds. More research also needs to be carried out on comparing the efficacy of phenolic compounds and extracts derived from medicinal and aromatic plants with that of the conventional fertilisers and pesticides. It has also been shown that these phenolic compounds and extracts can optimize the use efficiency of fertilisers and reinforce their effect thereby reducing the amounts of fertiliser needed. However, few studies exist, and further work needs to be done to establish conclusive evidence. Working towards wider acceptance and usage of these phenolic compounds and extracts from medicinal and aromatic plants as biostimulants and bioprotectants will require fine tuning to mineral fertilisers and pesticides and although challenging, this will be an important area of continued research.

A clear understanding of the above aspects will simplify the translation of these scientific studies into the industrial manufacture of viable biostimulant and bioprotectant formulations based on these phenolic compounds and extracts for use in agriculture with minimal challenges.

## Figures and Tables

**Table 1:** Compounds identified from selected medicinal and aromatic plants for the different phenolic classes.

Phenolic Compound	Phenolic Subcategory	Medicinal/Aromatic Plant	Reference
**Phenolic acids and derivatives**			
Syringic acid, gallic acid	Hydroxybenzoic acid	*Moringa oleifera*	[27]
Gallic acid, vanillic acid 3,4-dihydroxybenzoic acid, 4-hydroxybenzoic acid, syringic acid		*Peganum harmala*	[22]
Caffeic acid, *p*-coumaric acid	Hydroxycinnamic acid	*Origanum vulgare* L., *Salvia officinalis* L., *Thymus vulgaris* L., *Ocimum basilicum* L.	[28]
Vanillin	Hydroxybenzoic acid derivative	*T*. *vulgaris*	[29]
Rosmarinic acid	Hydroxycinnamic acid derivative	*Rosmarinus officinalis* L., *Mentha canadensis* L.	[28]
**Stilbenes**			
Piceatannol glucoside, resveratroloside, piceid	Stilbene glycosides	*Polygonum cuspidatum*	[30]
Trans-rhapontin, cis-rhapontin and trans-desoxyrhaponticin	Stilbene glycosides	*Rheum tanguticum* Maxim. ex Balf.	[31]
**Coumarins**			
Scopoletin, fraxetin, aesculetin, fraxin, aesculin	Coumarins, coumarin glycosides	*Fraxinus rhynchophylla.*	[32]
Herniarin	Coumarin	*Matricaria chamomilla*	[33]
Kayeassamin I, mammeasin E, mammeasin E	Geranylated coumarins	*Mammea siamensis*	[34]
**Lignans**			
Phyllanthin, hypophyllanthin, niranthin, nirtetralin, 5-demethoxy niranthin, virgatusin, heliobuphthalmin lactone and bursehernin	Lignans	*Phyllanthus amarus*	[35]
Schisanchinin A, schisanchinin B, schisanchinin C, schisanchinin D	Dibenzocyclooctadiene lignans	*Schisandra chinensis*	[36]
**Quinones**			
7-methlyjuglone	Naphthoquinone	*Drosera rotundifolia*	[37]
Aloe-emodin, emodin, chrysophanol, physcoin and rhein	Anthraquinones	*Rheumpalmatum* and *Rheum* *hotaoense*	[38]
**Curcuminoids**			
Curcumin, demethoxycurcumin and bis-demethoxycurcumin	Curcuminoids	*Curcuma longa*	[39]
**Flavonoids**			
Luteolin, apigenin, apigenin-O-glucuronide, luteolin-O-glycoside, orientin, apigenin-6,8-di-C-hexoside	Flavones and flavone glycosides	*Origanum majorana*	[40]
Daidzein, glycitein, genistein, formononetin, prunetin, biochanin A and daidzin, genistin	Isoflavone aglycones and isoflavone glycosides	*Medicago* spp.	[41]
Kaempferol 3-O-glucoside, isorhamnetin 3-O-galactoside	Flavonol glycosides	*Tephrosia vogelii*	[42]
Kaempferol 3-O-rhamnoside, rutin and quercetin 3-O-glucoside		*M.* *oleifera*	[27]
Naringenin-O-rhamnoglucoside, hesperidin, isosakuranetin-O-rutinoside	Flavanone glycosides	*Mentha pulegium*	[40]
Taxifolin, taxifolin methyl ether, dihydrokaempferide	Flavanonols	*O*. *majorana*	[40]
Gallocatechin, catechin	Flavan-3-ols	*M*. *pulegium*	[40]
**Tannins**			
Granatin A, punicalagin, peduncalagin I, ellagic acid, ellagic acid glucoside, ellagic acid pentoside, punigluconin	Ellagitannins	*Punica granatum*	[43]

**Table 2:** Total Phenolic Contents (TPC) and Total Flavonoid Contents (TFC) of selected medicinal and aromatic plants.

Medicinal/Aromatic Plant	Common Name	Plant Part	Solvent	Composition		Reference
				TPC	TFC	
*Acacia gourmaensis* ^1^		Stem bark	70% Ethanol	807.58	271.39	[75]
*Eclipta alba* ^1^	False daisy	Leaves	42.5 mL Methanol + 7.5 mL 1N HCl	55.32	31.55	[76]
		Roots		8.45	4.88	
		Whole plant	50% Ethanol	98.39	86.53	[73]
*Moringa oleifera* ^1^	Moringa	Leaves	Methanol	76.63	60.26	[77]
*Capparis spinosa* ^2^	Caper bush	Stem bark	80% Methanol	408.4	157.3	[64]
		Shoot		229.2	250.8	
		Fruit		535.8	283.3	
		Flowers		416.0	235.6	
		Roots		531.9	348.6	
*Coriandrum sativum* ^3^	Coriander	Seeds	70% Methanol	289.3	179.6	[61]
			Water	250.4	162.6	
*Thymus vulgaris* ^4^	Common thyme	Leaves	Water	256	44.2	[29]
			Ethanol	158	36.3	
*Cuminum cyminum* ^5^	Cumin	Seeds (Tunisian)	80% Acetone	18.60	5.91	[63]
		Seeds (Indian)		14.15	4.19	
*Cymbopogon citratus* (DC) Stapf. ^1^	Lemongrass	Leaves	Methanol	118.14		[62]
			Ethanol	35.43		
*Mentha pulegium* ^5^	Pennyroyal	Aerial parts	Methanol	18.28	13.76	[40]
*Peganum harmala* ^6^	African Rue	Seeds	75% Methanol	93.39	1.60	[78]

^1^ TPC values in mg GAE/g and TFC values in mg QE/g, ^2^ TPC in mg GAE 100 g^−1^ and TFC in mg CE 100 g^−1^, ^3^ TPC in mg GAE 100 g^−1^ and TFC in mg QE 100 g^−1^, ^4^ TPC in µg GAE/mg and TFC in µg QE/mg, ^5^ TPC values in mg GAE/g and TFC values in mg CE/g, ^6^ TPC values in g GAE/kg and TFC values in g QE/kg.

**Table 3:** Selected medicinal and aromatic plant extracts with biostimulant activity.

Medicinal/Aromatic Plant	Common Names	Plant Part	Application Method	Tested Concentrations	Biostimulated Parameters	Reference
*Acacia gourmaensis*		Bark	Seed immersion in extract	25% *w/v*	Seedling emergence, growth, vigour and weight in sorghum and pearl millet.	[110]
*Eclipta alba*	False daisy	Whole plant	Seed immersion in extract	25% *w/v*	Grain yield, seedling emergence, growth, vigour and weight in pearl millet and sorghum	[110]
*Moringa oleifera*	Moringa, horse-radish tree	Leaves	Foliar spray	1:32 *v/v* from 1000% *w/v* stock solution	Growth and yield in Cauliflower	[76]
		Leaves	Foliar spray	4%, 5%, ***6%*** from 10% *w/v* stock solution	Yield, colour, weight and firmness of plums	[111]
		Leaves and twigs	Foliar spray	1, ***2***, 3% leaf and 1, 2, ***3*%** twig extracts	Plant height, fresh herb weight and dry herb weight in rocket	[18]
*Crataegus monogyna* Jacq.	Hawthorn	Leaves		0.1, ***1.0*** mL/L of commercial extract	Dry weight of both roots and leaves of the maize plants	[19]
*Tephrosia vogelii*	Vogel’s tephrosia, fish-poison bean	Leaves	Foliar spray and soil drench	10% *w/v*	Plant height, number of leaves and branches, leaf area, stem width, leaf greenness in beans	[112]
		Leaves	Plant spraying	0.5, 2, ***5***% *w/v*	Yield (number of pods, pod length, shelled bean weight and seeds) in beans	[113]
		Leaves	Plant spraying	5, 10, ***20%*** from 50% *w/v* stock solution	Yield of watermelon	[114]
*Allium sativum*	Garlic	Clove	Seed soaking	***100***, 50, 25, 12.5% *w/v*	Percentage germination as well as root and shoot length of seedlings and plant vigour in rice	[115]
		Bulbs	Fertigation and foliar application	50, 100, 200 µg/mL	Plant height, leaf area, stem diameter, fresh and dry weight in tomatoes	[109]
*Zingiber officinale*	Ginger	Rhizome	Foliar application	***10*** and 15% from 50% *w/v* stock solution	Fruit yield in cucumber	[116]

The tested concentrations italicized in bold gave the highest biostimulant effect out of the tested concentrations.

**Table 4:** Selected phenolic compounds with bioprotectant activity.

Phenolic Compound	Solvent	Effective Concentration	Bioprotectant Effect	References
Ferulic acid	Water	LC_50_, 120 µg/mL	Strong repellent, nematistatic and nematicidal activity against *Radopholus similis*	[121]
Curcumin	95% Ethanol	MIC < 5 ppm	Inhibition of in vitro growth of *Clostridium perfringens*, *Clostridium butyricum* and *Clostridium sporogenes*	[122]
Ellagic acid	95% Ethanol	MIC < 5 ppm	Inhibition of in vitro growth of *Listeria monocytogenes*	[122]
Catechol, coumarin, gallic acid, caffeic acid, *p*-coumaric acid, ferulic acid	Water	MIC, 100–2000 µM	Inhibition of in vitro growth of *Xylella fastidiosa*	[123]
Catechin, naringenin, quercetin, resveratrol, rutin, sinapic acid	DMSO	MIC, 200–2000 µM	Inhibition of in vitro growth of *X. fastidiosa*	[123]
Salicylic acid		LC_50_, 46 µg/mL	Attractant, motility and hatch inhibition and nematicidal effects on *Meloidogyne incognita*	[121]
		2 mg/mL	Reduction of the severity of anthracnose disease caused by *Colletotrichum gloeosporioides* in mangoes	[124]
4-hydroxybenzoic acid	20% Methanol	0.1 to 1.5 mM	Herbicidal activity against *Dactylis glomerata*	[125]
Pyrogallol, ethylgallate, gentistic acid, juglone, apigenin 7-O-glucoside and 7-hydroxycoumarin	Water	1100 ppm	Nematicidal effects on *M. incognita*	[126,127]
Syringic acid	50% Acetone	1 mg/mL	Inhibition of radial growth of *Ganoderma boninense* in vitro	[128]

**Table 5:** Selected extracts from medicinal and aromatic plants with bioprotectant activity.

Medicinal/Aromatic Plant	Common Name	Plant Part	Extract Solvent	Effective Concentration	Bioprotectant Effect	References
*Acacia gourmaensis*		Bark	Water	25 g/100 mL	Reduced the incidence of *Phoma sorghina,* *Curvularia lunata* and *Fusarium moniliforme* in pearl millet seeds, and *Phoma sorghina* and *Colletotrichum graminicola* in sorghum seeds	[110]
*Azadirachta indica*	Neem tree	Leaf	Water	100 g/100 mL	Reduced incidence of seed borne *Bipolaris oryzae* in rice	[115]
*Cymbopogon citratus* (DC) Stapf.	Lemon grass	Leaves	Methanol	MIC, 25 μg/mL	Inhibition of in vitro growth of *Fusarium graminearum*	[62]
				MIC, 12.5 μg/mL	Inhibition of in vitro growth of *Fusarium oxysporum sp tulipae*	
*Thymbra spicata* L.	Black thyme, Mediterranean thyme	Leaves and flowers	Methanol	2%	Complete inhibition of in vitro growth of *Aspergillus parasiticus*	[129]
*Origanum compactum*		Aerial parts	Water, 80% methanol	25 g/L	Complete inhibition of radial growth of *Penicillium digitatum*	[130]
*Lantana camara* L.	Lantana, tickberry	Leaves	Water	25 g/100 mL	Complete inhibition of spore germination of *Curvularia tuberculata* and protected pear fruits from rot caused by the pathogen	[131]
				25 g/75 ml	Immobilised root knot nematodes, *M. incognita*	[132]
*Ocimum basilicum* L.	Basil	Leaves	Water	25 g/100 mL	Inhibition of spore germination of *Alternaria alternata* and protected pear fruits from rot caused by the pathogen	[131]
*Moringa olieifera*	Moringa, horse-radish tree	Leaves	Water	20% *v/v*	Reduced populations of adult *Phyllotreta cruciferae* insects in watermelon	[114]
*Tephrosia vogelii*	Vogel’s tephrosia, fish-poison bean	Leaves	Water	20% *v/v*	Reduced populations of adult *Diabrotica undecimpunctata* and *Dacus cucurbitea* insects in water mellon	[114]
				5% *w/v*	Reduction of aphid infestation in beans	[113]
*Mentha piperita* L.	Peppermint	Leaves and flowers	Water containing 0.05% Tween 80 and 4% ethanol	0.2%	Feeding deterrent of peach potato aphid, *Myzus persicae* and the bird-cherry oat aphid, *Rhopalosiphum padi* L. on pea and winter wheat; decreased fecundity and increased larval development time	[20]
*Echinacea angustifolia*	Narrow-leaf coneflower	Roots, aerial parts	Water	LC_50_ 352 µg/mL, 487 µg/mL	Caused juvenile mortality and inhibition of egg hatching in *M. incognita* nematodes	[21]
*Peganum harmala*	African Rue	Leaves, stems, roots	Water		Herbicidal activity against *Avena fatua* and *Convolvulus arvensis*	[22]

## Sample Availability

Samples of the compounds are available from the authors.
